# In Silico Hypothesis Testing in Drug Discovery: Using Quantitative Systems Pharmacology Modeling to Evaluate the Therapeutic Value of Proinsulin Conversion to Insulin Therapy for Type 2 Diabetes Mellitus

**DOI:** 10.3390/pharmaceutics17121522

**Published:** 2025-11-26

**Authors:** Maria E. Trujillo, Yue Han, Rebecca A. Baillie, Michael C. Weis, Douglas Chung, Sean Hayes, Paul E. Carrington, Michael Reed

**Affiliations:** 1 Merck & Co., Inc., Rahway, NJ 07065, USA; 2 Rosa and Co., LLC, San Carlos, CA 94070, USA

**Keywords:** quantitative systems pharmacology, type 2 diabetes, proinsulin, insulin, glucose

## Abstract

**Background/Objectives**: Proinsulin, the precursor to insulin, has limited activity on the insulin receptor. Proinsulin levels increase with increasing insulin resistance in type 2 diabetes due to incomplete processing by the β-cell. To assess whether the development of peptides that could convert circulating proinsulin to insulin in the blood would provide therapeutic value, we used a quantitative systems pharmacology (QSP) model of glucose homeostasis. In silico hypothesis testing such as this is an example of how modeling can inform decisions in drug discovery. **Methods**: In silico hypothesis testing involved (1) the addition and qualification of proinsulin biology into a preexisting QSP model, (2) the creation and validation of virtual patients (VPs) for subpopulations of type 2 diabetics based on phenotypic traits, and (3) the simulation of clinical trials evaluating the therapeutic value of the conversion of circulating proinsulin to insulin in the VPs created. **Results**: Proinsulin conversion led to a ~0.2% reduction in HbA1c in VPs at varying stages of diabetes, a decrease that does not hold meaningful therapeutic value. The lack of significant impact on HbA1c was likely a result of the surprisingly small effect on plasma insulin levels from proinsulin, which has a significantly slower secretion and clearance rate. Although patients with higher proinsulin/insulin ratios showed the largest reductions, clinically significant ≥ 0.5% reduction in HbA1c required ratios of proinsulin/insulin above the reported physiological range. **Conclusions**: This effort demonstrates how in silico hypothesis testing using QSP modeling can provide insights on the probability of success of novel interventions with minimal time and resources. These efficiencies are a means of overcoming the pressures on the pharmaceutical industry to do more with less in providing therapies that improve the lives of patients.

## 1. Introduction

Insulin secreted by pancreatic β-cells is a primary regulator of glucose levels in the fasted and postprandial states. Insulin synthesis includes post-translational processing in which proinsulin in the secretory vesicles of the β-cell is cleaved to form insulin and C-peptide. In healthy individuals, most proinsulin completes this process before secretion from the β-cell into the blood. In type 2 diabetes mellitus (T2DM), the processing pathways can become impaired, resulting in increased proinsulin secretion into the blood [1]. In addition, elevations in proinsulin are positively associated with insulin resistance [2,3,4,5,6,7]. In vitro studies show that proinsulin binding is ~10-fold less potent than insulin on the insulin receptor. These data suggest the increased proinsulin levels may introduce inefficiencies to the regulation of glucose by insulin in T2DM. Thus, it was hypothesized that correcting for the defect in proinsulin processing in T2DM by converting circulating proinsulin to insulin would increase circulating insulin, lower glucose, and ultimately reduce HBA1c.

Since less than ~50% of T2DM patients in the US achieve an HbA1c <7% [8], novel therapies (such as the conversion of proinsulin to insulin) that effectively lower glucose and HBA1c are of value. The current standard of care for T2DM includes diet, exercise, and treatment with either Glucagon Like Peptide-1 Receptor Agonists (GLP-1RAs) or Sodium Glucose Transporter 2 inhibitors (SGLT2is) [9]. GLP-1RAs reduce glucose and HBA1c by increasing insulin secretion and decreasing glucagon secretion in a glucose-dependent manner [10]. SGLT2is reduce glucose by increasing the urinary excretion of glucose [11]. Neither of these therapies address the defect in proinsulin processing or reduce the secretion of proinsulin. Thus, a putative therapeutic that converts circulating proinsulin to insulin should provide a novel therapeutic option for T2DM patients.

To evaluate the therapeutic potential of converting proinsulin to insulin, we performed experiments in silico using quantitative systems pharmacology (QSP) modeling. This approach uses mathematical models to describe the physiology of a system and the effects of drugs on various aspects of the system (i.e., pharmacology). Here we detail the features of a QSP model called the “Diabetes QSP model,” designed to capture the complexities of the glucose homeostatic system in human subjects that are healthy or T2DM. We also show how a “proinsulin sub-model” was added to the Diabetes QSP model to facilitate target identification/validation efforts in drug discovery.

The addition of the proinsulin sub-model was possible due to the many studies examining proinsulin and its role in diabetes pathophysiology, which also detailed the comparisons of proinsulin and insulin binding, potency, pharmacokinetics (PK), and pharmacodynamics [12]. The QSP modeling approach described herein provides an innovative way to address several questions about proinsulin that were otherwise difficult if not impossible to evaluate in the early drug discovery space. These questions included the following: (1) Could proinsulin conversion to insulin improve glycemia in T2DM? (2) Is there a subpopulation of T2DM patients that would most benefit from proinsulin to insulin conversion therapy?

The modeling and simulation work described resulted in the decision not to pursue the conversion of circulating proinsulin to insulin as an anti-diabetic therapy. This example of decision-making based on QSP modeling provides a strategic template for how others may leverage QSP to explore the potential of new therapeutic targets and facilitate the understanding of complex processes where data from multiple experiments and platforms converge via the model to render pivotal results. Importantly, this work was carried out with minimal experimental effort, resulting in significant resource savings.

## 2. Materials and Methods

A Diabetes QSP model validated by clinical data (Figure S1) was expanded to include a proinsulin sub-model using SimBiology (The MathWorks, MATLAB 2018b). Proinsulin and insulin concentrations from the literature and from samples analyzed from clinical trials detailed below were used to inform the proinsulin sub-model. Once the proinsulin sub-model was built and validated, virtual patients (VPs) with T2DM were created to test the therapeutic value of converting circulating proinsulin to insulin in plasma and its potential to lower HbA1c.

### 2.1. Diabetes QSP Model

**Figure 1:**
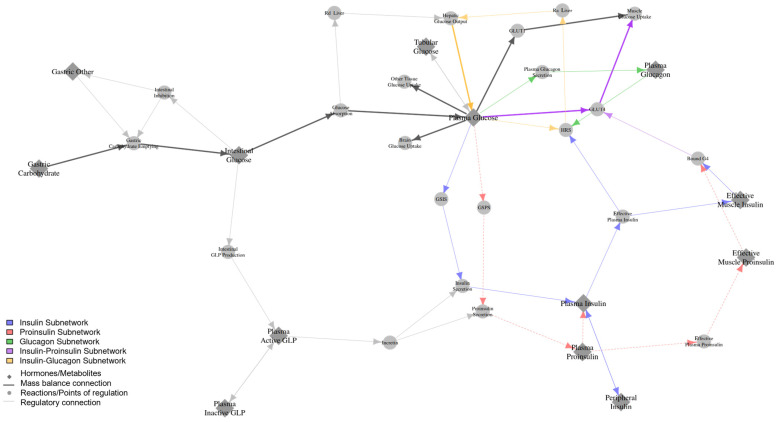
Schematic of the Diabetes QSP model and the proinsulin sub-model. Colored and gray nodes represent key hormones/metabolites and reactions/points of regulation, respectively. Gray arrows represent mass balance connections (bold) and regulatory connections (thin). The clearance and excretion reactions are omitted for clarity. Subnetworks for hormones, including proinsulin (dashed red arrow), insulin (blue arrow), glucagon (green arrow), insulin–proinsulin (purple arrow), and insulin–glucagon (yellow arrow), are highlighted in their respective colors.

The Diabetes QSP model is formulated as a set of non-linear ordinary differential equations representing plasma glucose regulation in both healthy individuals and those with T2DM. The dynamics of glucose, along with glucose-regulating hormones, including insulin, glucagon, and incretins, are described. The model tracks plasma glucose concentration and HbA1c level, which serve as diabetes biomarkers. Key equations from this model and the supporting literature are described below and in Figure 1. The complete set of equations, fluxes, and parameter values are found in the Supplementary Materials (Tables S1, S2 and S3, respectively). The model was validated through the generation of a virtual T2DM population treated with insulin glargine where the simulated time-course of HbA1c and plasma glucose concentration aligns well with clinical data from Zinman et al. (Figure S1) [13].

In the model, carbohydrates may be introduced into the system through the ingestion of a meal. The gastric carbohydrate emptying rate is determined by meal content and feedback from the GI tract [14]. The absorption of glucose from the GI tract to plasma is assumed to be proportional to the intestinal glucose levels. These assumptions are supported by high-quality data in humans [15,16]. In addition to the absorption from the GI tract, plasma glucose is supplied by liver glucose production and taken up by liver, muscle, brain, and other tissues (Equation (1)).

d(PlasmaGlucose)/dt = GlucoseAbsorption + HepaticGlucoseOutput − MuscleGlucoseUptake − BrainGlucoseUptake − OtherTissueGlucoseUptake − GlucosePlasmaToTubular(1)

The supply of glucose to plasma by liver is regulated by glucose, insulin, and glucagon [17,18], and the uptake of glucose by liver assumes a basal rate plus a term proportional to the absorption rate of glucose from GI. The exchange of glucose between plasma and liver is represented as a hepatic glucose output term. Muscle glucose uptake is represented as a summed value from GLUT1 and GLUT4 transporters, with GLUT1 being independent of insulin and GLUT4 highly responsive to muscle insulin levels [19]. Brain glucose uptake is assumed to be roughly constant, while a pool of “other tissues” (e.g., skin, red blood cells, adipose tissue) are assumed to take up glucose at a rate proportional to plasma glucose concentration [20,21,22].

In addition to the removal of glucose from plasma by tissues that metabolize glucose as a fuel source, the rate of glucose clearance from the plasma is influenced by the process of renal filtration. Glucose is filtered from the plasma by the kidneys and then re-absorbed. Virtual subjects with hyperglycemia (as in type 2 diabetes) display incomplete re-absorption, leading to urinary glucose excretion [23,24]. Urinary glucose excretion (UGE) is assumed to be proportional to plasma glucose levels above a renal set threshold [25].

As mentioned above, tissues such as liver and muscle affect plasma glucose in ways that are dependent on plasma insulin level (Equation (2)).

d(PlasmaInsulin)/dt = InsulinSecretion + InsulinPeriphToPlasma − InsulinClearance(2)

Insulin is generated in the pancreas via glucose-stimulated insulin secretion regulated by the active plasma GLP-1 level and its distribution and clearance is assumed to follow a 2-compartment PK model [26]. GLP-1 is an incretin that when secreted enhances the secretion of insulin. Plasma GLP-1 is assumed to be produced at a constant baseline rate plus at an increased rate in response to the intestinal glucose level. Circulating GLP-1 is deactivated by endogenous dipeptidyl peptidase-4 (DPP-4) and other enzymes (Equation (3)).

d(PlasmaActiveGLP)/dt = Basal_GLP + IntestinalGLPProduction − GLPInactivationDPP4 − GLPInactivationGLP(3)

Glucagon is another hormone produced by the pancreas that contributes to regulating plasma glucose levels (Equation (4)).

d(PlasmaGlucagon)/dt = PlasmaGlucagonSecretion − PlasmaGlucagonClearance(4)

Glucagon secretion is represented such that for healthy individuals, there is a basal rate and a Hill-type equation based on plasma glucose. For T2DM individuals, glucagon secretion is compromised, and a parameter is used to represent the degree of dysregulation of glucagon.

### 2.2. Proinsulin Sub-Model

The proinsulin sub-model includes proinsulin production, degradation, and conversion to insulin, as described in Equation (5) (Figure 1). Since proinsulin is converted to insulin in the plasma, the “ProinsulinConv” term in Equation (5) is added to the plasma insulin mass balance described in Equation (2).

d(PlasmaProinsulin)/dt = ProinsulinSecretion − ProinsulinConv − ProinsulinClearance(5)

Proinsulin is co-secreted with insulin; thus, regulation of proinsulin secretion was assumed to be the same as insulin secretion. Proinsulin secretion is affected by glucose levels (Glucose-Stimulated Proinsulin Secretion, GSPS) and incretins such as GLP-1. The proportion of proinsulin secreted with insulin increases in VPs with insulin resistance or T2DM [27,28,29,30,31,32,33,34,35,36]. Proinsulin kinetics were based on the following key assumptions: (1) under fasting conditions, kinetic parameters must maintain the ratio of proinsulin to insulin ~0.2 in the systemic circulation observed under fasting conditions [29,31,32,33,34,35]; (2) the half-life of insulin is 5–10-fold shorter than proinsulin [37,38,39,40,41]; and (3) the volume of distribution for proinsulin and insulin are the same.

To explore the potential anti-diabetic effects of a hypothetical proinsulin-converting drug, a first-order rate equation to increase the rate of proinsulin conversion to insulin in the plasma was added to the model. The effect of treatment with this hypothetical drug on insulin and proinsulin is described in Equation (6) where K_Cpro is the rate of conversion. Explorations of conversion rates up to ~7 pM/min were conducted through simulations of OGTTs. The rate of ~7pM/min was eventually chosen based on these results (see Section 2.6 for additional details). Proinsulin increases glucose uptake by muscle via the GLUT4 transporter, although to a lesser extent than insulin [42,43,44].

ProinsulinConv = 1/K_Cpro × PlasmaProinsulin(6)

### 2.3. Clinical Data Informs the Proinsulin Sub-Model

Proinsulin was measured from samples acquired from two completed Phase 3 studies conducted in participants with T2DM (Study 11, NCT01717313; and Study 24, NCT01755156). Additional information on the studies may be found at clinicaltrials.gov. Three hundred and seven subjects from these studies had both baseline clinical data and consented samples stored for future biomedical research. Samples from all subjects prior to treatment were used for the measurement of insulin and proinsulin.

### 2.4. Proinsulin and Insulin Measurement in Clinical Samples

Measurements of proinsulin and insulin were performed using commercially available immunoassays selective for each protein (Proinsulin, Cat. No. 10-1118 and Insulin Cat. No. 10-1113; Mercodia, Uppsala, Sweden). To facilitate direct comparisons of insulin and proinsulin concentrations in clinical samples, calibrators with accurate molar concentrations as determined by amino acid analysis were used.

### 2.5. Virtual Patients

Given the heterogeneity of T2DM, three separate diabetic virtual patients (VPs) were created to facilitate the evaluation of proinsulin to insulin conversion therapy. Each VP was meant to be representative of a phenotype that may be present within the spectrum of T2DM. In the early and middle stages of diabetes progression, captured by VPs VPT2DM-1 and VPT2DM-2, these alterations include increasing hyperinsulinemia, hyperproinsulinemia, and hyperglycemia caused by increasing insulin resistance and compensatory insulin secretion from functioning pancreatic beta cells. In late-stage diabetes, captured by VPT2DM-3, hyperglycemia is more pronounced and there is a decline in insulin secretion due to β-cell failure. In this phenotype, there is an increased insulin resistance and significant β-cell defects, resulting in decreased insulin and proinsulin secretion. These VPs were created by introducing changes into the model parameters that are reflective of the known perturbations in the pathophysiology of T2DM including (1) insulin resistance (i.e., decreased sensitivity of glucose output by the liver to regulation by insulin, glucagon and glucose; and an increase in the Km of glucose uptake by the muscle) resulting in hyperglycemia, and (2) β-cell defects (i.e., a reduced Vmax and increased Km for glucose-stimulated insulin and proinsulin secretion) resulting in reduced insulin and proinsulin secretion with advanced diabetes. Changes to the model parameters used in the construction of these VPs are found in Table S3. Information on glucose, insulin, and HbA1c expected in these VPs are provided (Table 1). Information on a healthy participant (VPHealthy) is also provided for comparison.

### 2.6. Simulations of Proinsulin to Insulin Conversion Therapy

To examine the performance of the proinsulin sub-model, three meals a day were administered to VPs that were then treated or not treated with proinsulin to insulin conversion therapy for one year; HbA1c was reported. At the end of one year of treatment, an oral glucose tolerance test (OGTT) was simulated whereby each VP was fasted for 16 h and then presented with an oral glucose load of 75 g. Resulting changes in glucose, insulin, and proinsulin over time are reported.

To test the therapeutic potential of proinsulin conversion to insulin, conversion rates of up to ~7 pM/min were explored. Since this therapy is assumed to exhibit enzymatic properties, this rate includes a delay to more closely approximate the timing of protein binding and cleavage. Preliminary results demonstrated that ~7 pM/min conversion rate resulted in a significant reduction in proinsulin levels during an OGTT and was therefore employed in the remainder of the simulations. This conversion rate resulted in nearly complete removal of proinsulin in the fasting and late post prandial state in all virtual patients (Figure S2). Thus, higher levels of proinsulin conversion were not explored.

## 3. Results

### 3.1. Proinsulin Sub-Model Validation

The proinsulin sub-model was validated both qualitatively and quantitatively. First, fasting insulin, proinsulin, and the ratio of proinsulin to insulin in VPs was compared to data in the literature as well as that measured from baseline samples from previously conducted clinical trials. The values of insulin and proinsulin were consistent with the clinical data previously reported and observed, supporting the VPs. The proinsulin sub-model produced plausible representations of participants with T2DM (Table 2).

**Figure 2:**
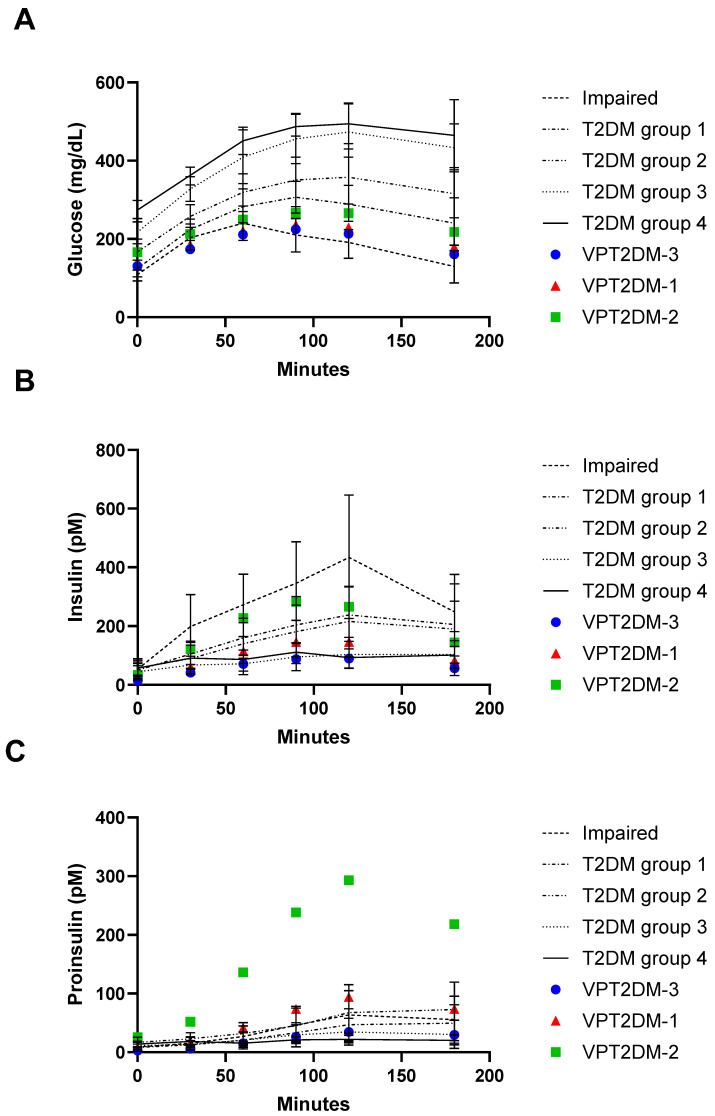
Changes during an oral glucose tolerance test are presented for (**A**) glucose, (**B**) insulin, and (**C**) proinsulin. Data from Yoshioka et al. 1988 [27] from participants that were of increasingly impaired glucose tolerance to T2DM (groups 1–4) are represented by solid lines (representing means and standard deviations). Data from simulations of VPs with moderate T2DM (VPT2DM-1 [red triangle], VPT2DM-2, [green square]) and more severe T2DM (VPT2DM-3, [blue circle]) at individual time points are overlaid for comparison.

In addition to static observations, dynamic changes in insulin and proinsulin were also used to evaluate the proinsulin sub-model. The results from simulations of an OGTT for each VP were compared with those reported by Yoshioka et al. ([27]; Figure 2). Simulations of changes in glucose, insulin, and proinsulin during an OGTT in T2DM VPs were found to be each of the appropriate level and duration, reflecting the differences in pathophysiology that can be expected at different stages of diabetes. The OGTT results from these three VPs further validated the proinsulin sub-model and the utility of these VPs in testing anti-diabetic therapies. To provide information on patients with above average proinsulin levels, one of the VPs (VPT2DM-2) was engineered to have severe hyper-proinsulinemia. In diabetics, proinsulin levels up to 500 pM have been reported during an oral glucose challenge [27].

### 3.2. Evaluations of Proinsulin Conversion to Insulin Therapy

**Figure 3:**
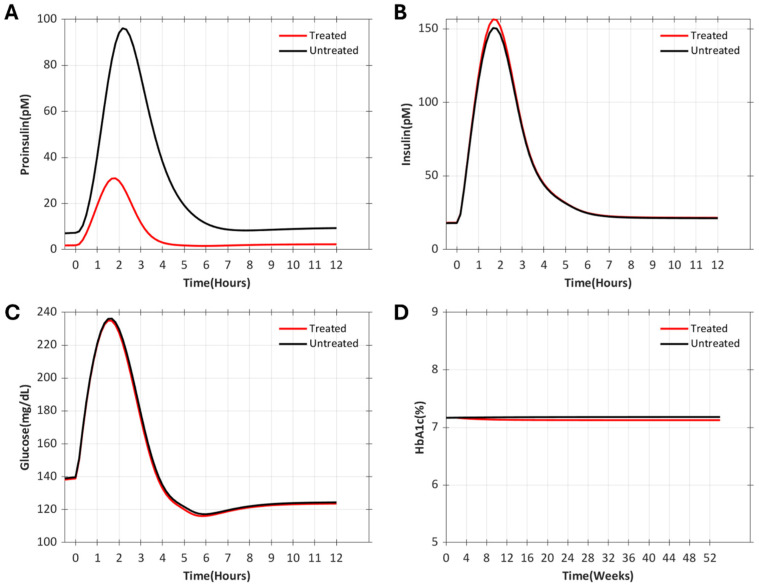
Simulation of therapy converting proinsulin to insulin for 52 weeks of treatment in a VP with moderate T2DM and higher than average proinsulin levels (VPT2DM-2). Assessments of proinsulin (**A**), insulin (**B**), and glucose (**C**) were made during an OGTT administered at the end of treatment. (**D**) HbA1c levels were assessed throughout the treatment period.

After one year of simulated proinsulin conversion to insulin therapy, T2DM VPs that were fed three meals each day were assessed for changes in glucose, insulin, and proinsulin, following an OGTT on the last day of the trial, and for changes in HbA1c throughout the duration of the trial. The results of these simulations demonstrated a reduction in proinsulin levels without clinically meaningful changes in insulin, glucose, or HbA1c in T2DM VPs (data from VPT2DM-2 shown in Figure 3).

**Figure 4:**
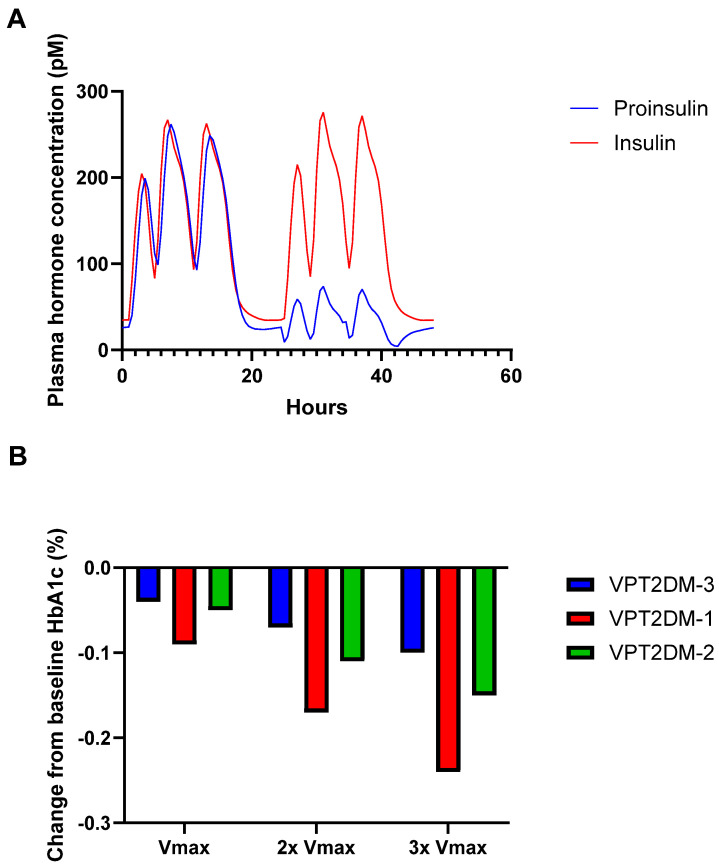
(**A**) Results from simulations of changes in insulin and proinsulin levels 24 h before and after treatment with proinsulin conversion to insulin therapy in VPT2DM-1; and (**B**) effect of increasing proinsulin secretion on HbA1c in each VP (VPT2DM-1, VPT2DM-2, and VPT2DM-3) treated with proinsulin conversion therapy.

Additional simulations were performed to confirm the proinsulin to insulin conversion therapy resulted in desired changes to insulin/proinsulin levels. Simulation results for insulin and proinsulin over the course of a day with the administration of three meals showed that the conversion of proinsulin to insulin immediately decreased proinsulin levels (Figure 4A). In contrast, insulin levels increase by ~10 pM at the peak of secretion (after a meal). These results demonstrate that proinsulin conversion successfully increased insulin levels, but the resultant increase in insulin was small and ineffective, even in the VP with the highest proinsulin secretion rate (VPT2DM-2). While a significant amount of proinsulin is converted (as shown in Figure 3A and Figure 4A), the insulin produced is quickly cleared without accumulation. This is likely due to the fast clearance rate of insulin relative to the secretion and conversion of proinsulin.

Finally, it was hypothesized that proinsulin to insulin conversion therapy may only be effective in VPs with even higher proinsulin secretion levels. To test this hypothesis, simulations of proinsulin therapy over the course of one year in VPs administered three meals daily were conducted. Virtual patients with increased baseline proinsulin/insulin ratios were created by increasing the Vmax for proinsulin secretion (Vmax_GSPS) from 0.2 in the base model to 0.4 (2×) or 0.6 (3×). Initial proinsulin to insulin ratios were in the range of 0.23–0.73 (Table 2, baseline levels). With the increase in Vmax for proinsulin secretion, the proinsulin/insulin ratios increased 2–3-fold, resulting in proinsulin to insulin ratios ranging from 0.53 to 2.33 across the different VPs. Indeed, increasing proinsulin secretion increased the ratios of proinsulin/insulin. Treatment of such VPs resulted in an increased effectiveness of proinsulin to insulin conversion therapy (Figure 4B); however, the effect was small (≤0.25% change in HbA1c). It should be noted that changing the Vmax by 3-fold or more results in proinsulin/insulin ratios above those commonly observed in clinical trials (Table 2, reference data).

## 4. Discussion

Here we share our understanding of the regulation of glucose homeostasis represented in a Diabetes QSP model and the process of how our Diabetes QSP model was expanded to incorporate data relevant to the biology of proinsulin from the literature. We then describe how the proinsulin sub-model was utilized for hypothesis testing in silico to determine whether a putative therapy that converts circulating proinsulin to insulin benefits T2DM patients. The modeling and simulation work described resulted in the decision not to pursue the conversion of circulating proinsulin to insulin as an anti-diabetic therapy. This example of how existing data can be leveraged via a model to test whether putative therapies resolve β-cell deficiencies is a very efficient and effective way to triage drug discovery before investing in expensive programs.

To evaluate the therapeutic value of the conversion of proinsulin to insulin in T2DM, a QSP model was chosen because it best captures the complexities of the physiology of glucose homeostasis and its derangement in T2DM. Multiple tissues and hormones, including insulin, converge to regulate glucose acutely (hours) and HbA1c over extended periods (months). Simpler models capture the acute effects of changes in insulin on glucose, such as the minimal model [49] and the integrated glucose insulin model [50]. However, neither of these models facilitates the breadth of understanding and mechanistic insights provided by QSP modeling. For example, initial simulations of proinsulin to insulin conversion therapy showed no effect on glucose or HbA1c. Additional exploration with the proinsulin sub-model confirmed that proinsulin responded to stimuli such as meals and other glycemic challenges as expected. Research explorations such as these are well suited to interrogation by QSP.

A key and somewhat counterintuitive finding of the modeling was that the conversion of circulating proinsulin to insulin had a small, short-lived increase in circulating insulin that was not accompanied by glycemic benefit. This result was surprising because proinsulin levels are increased in diabetes and proinsulin is ~10-fold less potent on the insulin receptor, potentially contributing to the diabetic state. Therefore, it was hypothesized that the conversion of proinsulin to insulin could correct this defect and improve glucose control. Furthermore, the conversion of the levels of proinsulin observed in plasma (1:1 on a stoichiometric basis) should increase insulin to levels within the dynamic range for glucose uptake by skeletal muscle [51,52], the primary source of insulin- stimulated glucose disposal. Together, these data suggest that the conversion of proinsulin to insulin could result in a meaningful reduction in circulating glucose in T2DM. However, the simulated results showed no improvement in glycemia with proinsulin conversion.

Closer examination of the simulated results revealed that the insulin produced from the conversion of proinsulin was rapidly cleared, removing the potential for a change in glycemia. This result is likely due to the very different secretion and clearance rates for proinsulin and insulin. Proinsulin is known to have much slower secretion and clearance rates compared to insulin [38,39,41,53,54,55]. These features result in a proinsulin pool where the turnover is much slower than that of insulin. Therefore, when circulating proinsulin is converted to insulin, it enters a pool of much more rapid turnover, is rapidly cleared, and does not provide a meaningful source of additional insulin. This finding was only made evident through dynamic simulations using the QSP model. Further, this lack of effect was consistent across the range of virtual diabetic patients differing in circulating insulin/proinsulin and insulin resistance observed in the heterogeneous T2DM population.

Sensitivity analyses exploring increases in proinsulin secretion resulting in proinsulin/insulin ratios as high as 3:1 within our diabetic VPs was conducted. Treatment of these VPs with conversion therapy resulted in modest changes in HbA1c (−0.25% change in HbA1c from baseline). However, because proinsulin/insulin ratios above 2 are rare [29,32,33,34,35,45], and less than 1% of individuals in our studies meet this criterion (Table 2), the subpopulation of diabetics that could benefit from the proposed therapy is likely few. In addition, the resulting changes in HBA1c (~0.25% with proinsulin conversion) in these individuals are small compared to the standard of care, such as GLP-1RA and SGLT2i, where changes in HBA1c can be 1.6% and 0.9%, respectively [8,9,10,11]. Therefore, the therapeutic benefit against comparators is limited.

Though informative and impactful, the work described in this manuscript had several limitations. The greatest limitation to our work was that this study tested a hypothesis only in silico.

Another key limitation of this study was that although the proinsulin conversion rates tested reduced fasting and late postprandial insulin levels to near zero, a maximum conversion rate that eliminated circulating proinsulin was not achieved. The maximum rate of proinsulin conversion tested (~7 pmol/min) was set to be far higher than the fasting proinsulin secretion rate in any of the VPs and higher than the maximum proinsulin conversion in the base model. However, in the diabetic VPs, the postprandial rate of proinsulin (and insulin) secretion increases dramatically during an OGTT such that a small transient amount of proinsulin remained. Though it is possible that there could be better efficacy with conversion rates higher than those tested, increasing rates of proinsulin conversion resulted in increasingly incremental improvements in HBA1c, suggesting that testing higher rates of conversion were unlikely to change the conclusion.

An additional limitation of this study is that non-glycemic effects of the conversion of proinsulin to insulin are not evaluated by the current Proinsulin QSP model. One potential missed benefit is the removal of autoreactive proinsulin. Proinsulin is identified as one of the major autoantigens involved in the pathogenesis of T1D that leads to T-cell-mediated β-cell destruction [56,57]. In T2DM, patients diagnosed with latent autoimmune diabetes of adults have detectable proinsulin autoantigens [58,59]. Lastly, while not proving a direct cause, studies have shown that elevated serum proinsulin concentrations are an independent predictor for cardiovascular disease risk [60,61]. Select diabetic patients may therefore benefit from reduced morbidity and mortality from a therapy that removes circulating proinsulin. The evaluation of non-glycemic benefits such as the removal of autoreactive proinsulin is beyond the scope of the current model.

## 5. Conclusions

QSP modeling is a valuable tool for in silico hypothesis testing that can facilitate early decisions in drug discovery. The example described here uses QSP modeling to test the potential therapeutic value of converting circulating proinsulin to insulin in T2DM patients based on readily interpretable clinical endpoints, i.e., changes in glucose or HbA1c. The simulations presented here show that proinsulin to insulin conversion therapy would not likely provide meaningful clinical benefit in any subpopulation of T2DM (VPs) explored. This hypothesis would otherwise be difficult, if not impossible, to address without significant investment in experimental research and development. Further, these simulations provided mechanistic insight for the lack of efficacy with the proposed therapy.

Future efforts to create and leverage QSP models in various therapeutic areas in drug discovery and development are ongoing [62,63]. Scientists of diverse perspectives may use QSP models as a framework to integrate data to address research questions and develop novel therapies that improve the lives of patients.

## Figures and Tables

**Table 1:** Baseline characteristics of a healthy participant and T2DM VPs created with the Proinsulin QSP model.

Virtual Patient	VPHealthy	VPT2DM-1	VPT2DM-2	VPT2DM-3
Disease Diagnosis	Healthy	Moderate	Moderate	Severe
Patient Type Representation	Healthy VP with low insulin secretion and very high insulin sensitivity	Early-stage type 2 diabetes with high insulin resistance and pancreatic compensation	Early-stage type 2 diabetes with high insulin resistance and hyperinsulinemia	Late-stage type 2 diabetes with pancreatic failure
Fasting glucose (mg/dL)	93	140	131	167
Fasting insulin, (pM)	28	17	34	13
Postprandial glucose (mg/dL)	152	209	196	240
Postprandial insulin (pM)	180	150	270	110
HbA1c (%)	4.9	7.2	6.6	8.2

**Table 2:** Baseline characteristics of a healthy participant and T2DM VPs created with the Proinsulin QSP model. For reference data, mean values and ranges are shown.

	Disease Status	Insulin, pM	Proinsulin, pM	Proinsulin/Insulin
Reference Data				
Ave. lit. data ^1^	T2DM	92 (41–370)	19 (6–51)	0.21
Ave. lit. data ^2^	Healthy	56 (21–148)	7 (3–13)	0.12
Study 11	T2DM (n = 181)	104 (13–295)	34 (8–173)	0.31 (0.14–0.68)
Study 24	T2DM (n = 127)	133 (5–1120)	44 (2–263)	0.44 (0.03–4.81)
Model Prediction				
VPHealthy	Healthy	28	5	0.18
VPT2DM-1	Moderate T2DM	17	7	0.41
VPT2DM-2	Moderate T2DM	34	25	0.73
VPT2DM-3	Severe T2DM	13	3	0.23

^1^ [27,28,29,31,32,33,35,36,45]; ^2^ [27,28,29,31,36,45,46,47,48].

## Data Availability

The original contributions presented in this study are included in the article/Supplementary Materials except for clinical data. Requests for access to the clinical study data can be submitted through the EngageZone site or via email to dataaccess@merck.com. The data sharing policy related to these clinical data, including restrictions, of Merck Sharp & Dohme LLC, a subsidiary of Merck & Co., Inc., Rahway, NJ, USA, is available at https://externaldatasharing-msd.com/ (accessed on 18 November 2025).

## Supplementary Materials

The following supporting information can be downloaded at: https://www.mdpi.com/article/10.3390/pharmaceutics17121522/s1. Figure S1: Validation of Diabetes QSP model with clinical data; Figure S2. Simulated plasma proinsulin concentration assuming varying proinsulin conversion rates during an OGTT in virtual patients; Table S1: Ordinary differential equations (ODEs) in the Diabetes QSP model including the proinsulin sub-model; Table S2: List of fluxes and rules in the Diabetes QSP model including the proinsulin sub-model; Table S3: List of parameters in the Diabetes QSP model including the proinsulin sub-model. Table S4: IRB approval number and dates Study #11; Table S5: IRB approval number and dates Study #24.

## Abbreviations

The following abbreviations are used in this manuscript:

QSP	Quantitative systems pharmacology
VP	Virtual patient
HbA1c	Glycated hemoglobin
T2DM	Type-2 diabetes mellitus
UGE	Urinary glucose excretion
GLP-1	Glucagon-like peptide-1
GSPS	Glucose-stimulated proinsulin secretion
OGTT	Oral glucose tolerance test
PK	Pharmacokinetics
