# Supplementary material for: In Silico Hypothesis Testing in Drug Discovery: Using Quantitative Systems Pharmacology Modeling to Evaluate the Therapeutic Value of Proinsulin Conversion to Insulin Therapy for Type 2 Diabetes Mellitus
Source: Pharmaceutics. 2025 Nov 26;17(12):1522. doi: 10.3390/pharmaceutics17121522 (PMC12736522; doi:10.3390/pharmaceutics17121522)
Supplement: Supplementary file 1 [file pharmaceutics-17-01522-s001.zip › pharmaceutics-3933941-supplementary.pdf]

Supplementary Material

In silico Hypothesis Testing in Drug Discovery: Using Quantitative Systems Pharmacology Modeling to Evaluate the Therapeutic Value of Proinsulin Conversion to Insulin Therapy for Type 2 Diabetes Mellitus

Maria E. Trujillo, Yue Han, Rebecca A. Baillie, Sean Hayes, Michael C. Weis, Douglas Chung, Paul E. Carrington, Michael Reed

Supplementary Table S1. Ordinary differential equations (ODE) in the Diabetes QSP model including the Proinsulin Sub-model.

**Ordinary differential Equations**
d(GastricCarbohydrate)/dt = FoodCarbonhydrateIntake - GastricCarbohydrateEmptying
d(GastricOther)/dt = FoodOtherIntake - GastricOtherEmptying
d(IntestinalGlucose)/dt = 250 * GastricCarbohydrateEmptying - GlucoseAbsorption
d(PlasmaGlucose)/dt = GlucoseAbsorption + HepaticGlucoseOutput - MuscleGlucoseUptake - BrainGlucoseUptake - OtherTissueGlucoseUptake - GlucosePlasmaToTubular
d(TubularGlucose)/dt = GlucosePlasmaToTubular - TubularGlucoseExcretion
d(PlasmaInsulin)/dt = InsulinSecretion + InsulinPeriphToPlasma - InsulinClearance + ProinsulinConv
d(PeripheralInsulin)/dt = - InsulinPeriphToPlasma
d(PlasmaActiveGLP)/dt = Basal_GLP + IntestinalGLPProduction - GLPInactivationByDPP4 – GLPInactivationByGLP
d(PlasmaInactiveGLP)/dt = GLPInactivationByDPP4 + GLPInactivationByGLP - InactiveGLPClearance
d(PlasmaGlucagon)/dt = PlasmaGlucagonSecretion - PlasmaGlucagonClearance
d(Glucose30DayDelay)/dt = (Conc_Glucose_Pl - Glucose30DayDelay)/43200
d(Glucose90DayDelay)/dt = (Conc_Glucose_Pl - Glucose90DayDelay)/129600
d(PlasmaProinsulin)/dt = ProinsulinSecretion– ProinsulinConv – ProinsulinClearance
d(EffectiveMuscleInsulin)/dt = InsulinEquilibrium
d(EffectiveMuscleProinsulin)/dt =ProinsulinEquilibrium

Supplementary Table S2. List of fluxes and rules in the Diabetes QSP model including the Proinsulin Sub-model.

A1c = 1.4+(0.7* Glucose30DayDelay +0.3* Glucose90DayDelay)/28
Conc_Glucose_Pl = PlasmaGlucose/Vd_PlasmaGlucose
Conc_Insulin_Pl = PlasmaInsulin/V_Insulin
Conc_Proinsulin_Pl = PlasmaProinsulin/V_Proinsulin
EffectivePlasmaInsulin = 1000 * PlasmaInsulin / (6 * V_Insulin)
EffectivePlasmaProinsulin = 1000 * PlasmaProinsulin / (6 * V_Proinsulin)
GastricTotal = GastricCarbohydrate + GastricOther
GastricCarbohydrateEmptying = IntestinalInhibition * Vmax_GE * GastricCarbohydrate / (Km_GE + GastricTotal)
GastricOtherEmptying = IntestinalInhibition*Vmax_GE*GastricOther/(Km_GE+GastricTotal)
IntestinalInhibition = 1 - IntestinalGlucose / (Km_GEi + IntestinalGlucose)
GlucoseAbsorption = K_Absorption*IntestinalGlucose
HepaticGlucoseOutput = Ra_Liver - Rd_Liver
Rd_Liver = Basal_GK + Ka_GK * GlucoseAbsorption
Ra_Liver = Vmax_G6P/(HRS*Km_Ra_Liver+1)
HRS = ((Conc_Glucose_Pl/NormalGlucose)*(EffectivePlasmaInsulin/NormalInsulin))/(PlasmaGlucagon/NormalGlucagon)
MuscleGlucoseUptake = GLUT1 + GLUT4
GLUT1 = Vmax_MG1*Conc_Glucose_Pl/(Km_MG1+Conc_Glucose_Pl)
GLUT4 = BoundG4*Vmax_MG4*Conc_Glucose_Pl/(Km_MG4+Conc_Glucose_Pl)
BoundG4 = EffectiveMuscleInsulin^h_MIns/(Km_Ins_GLUT4^h_MIns+EffectiveMuscleInsulin^h_MIns)+EffectiveMuscleProinsulin^h_MIns/(Km_Pro_GLUT4^h_MIns+EffectiveMuscleProinsulin^h_MIns)
InsulinEquilibrium = (1/K_MIns) * (EffectivePlasmaInsulin - EffectiveMuscleInsulin)
GlucosePlasmaToTubular = Filtration-Reabsorption
Filtration = Ka_TubGlu*Conc_Glucose_Pl
Reabsorption = TmG * TubularGlucose / (Km_UGR + TubularGlucose)
BrainGlucoseUptake = Vmax_Brain*Conc_Glucose_Pl/(Km_Brain+Conc_Glucose_Pl)
OtherTissueGlucoseUptake = Vmax_OT*Conc_Glucose_Pl/(Km_OT+Conc_Glucose_Pl)
TubularGlucoseExcretion = Ka_UGE*TubularGlucose
InsulinPeriphToPlasma = PeripheralInsulin*Q_Insulin/VP_Insulin-PlasmaInsulin*Q_Insulin/V_Insulin
InsulinClearance = PlasmaInsulin*CL_Insulin/V_Insulin
InsulinSecretion = GSIS * Incretin
GSIS = Vmax_GSIS *Conc_Glucose_Pl^4/(Km_GSIS ^4+Conc_Glucose_Pl^4)
GSPS = Vmax_GSPS*Conc_Glucose_Pl^n_GSPS/(Km_GSPS ^n_GSPS+Conc_Glucose_Pl^n_GSPS)

Incretin = Vmax_Incretin * PlasmaActiveGLP / (Km_Incretin + PlasmaActiveGLP)
IntestinalGLPProduction = Ka_aGLP * IntestinalGlucose
GLPInactivationByDPP4 = Kd_DPP4 * PlasmaActiveGLP
GLPInactivationByGLP = Kd_aGLP * PlasmaActiveGLP
InactiveGLPClearance = Kd_iGLP * PlasmaInactiveGLP
PlasmaGlucagonSecretion = normal_BasalGGN+defective_BasalGGN*(1-afx)/(Km_defective_BasalGGN+(1-afx))+normal_Vm_GGN*afx/((Km_GGN^4)*(Conc_Glucose_Pl^4)+1)
PlasmaGlucagonClearance = PlasmaGlucagon / 15
ProinsulinConv = 1/K_CPro*PlasmaProinsulin
ProinsulinSecretion = GSPS * Incretin
ProinsulinEqulibrium = (1/K_MPro)*(EffectivePlasmaProinsulin-EffectiveMuscleProinsulin)
ProinsulinClearance = PlasmaProinsulin * CL_Proinsulin / V_Proinsulin

Supplementary Table S3. List of parameters in the Diabetes QSP model including the Proinsulin Sub-model. Parameters with different values in various virtual patients are highlighted, where a darker color indicate a larger value.

Name	VPHealthy	VPT2DM-1	VPT2DM-2	VPT2DM-3
Vd_PlasmaGlucose	140	140	140	140
CL_Proinsulin	0.084	0.084	0.084	0.084
V_Proinsulin	5.11	5.11	5.11	5.11
V_Insulin	5.11	5.11	5.11	5.11
VP_Insulin	31.6	31.6	31.6	31.6
Q_Insulin	0.4083	0.4083	0.4083	0.4083
CL_Insulin	0.7283	0.7283	0.7283	0.7283
Vmax_GE	8	8	8	8
Km_GE	100	100	100	100
Km_GEi	4000	4000	4000	4000
K_Absorption	0.1	0.1	0.1	0.1
Basal_GK	5	5	5	5
NormalGlucose	90	90	90	90
NormalInsulin	5	5	5	5
NormalGlucagon	75	75	75	75
Ka_GK	0.3	0.3	0.3	0.3
Km_Ra_Liver	3	1.5	2.4	2.1
Vmax_MG1	24	24	24	24
Km_MG1	18	18	18	18
Vmax_MG4	1800	1800	1800	1800
Km_MG4	180	180	180	180
Km_Pro_GLUT4	5187.19	10374.38	6483.9875	7410.271
Km_Ins_GLUT4	37.6137	75.2274	47.017125	53.73386
Km_MIns	37.6137	37.6137	37.6137	37.6137
h_MIns	3.0612	3.0612	3.0612	3.0612
K_MIns	30	30	30	30
Ka_TubGlu	1.13	1.13	1.13	1.13
Km_UGR	100	100	100	100
TmG	180	180	180	180
Ka_UGE	0.01	0.01	0.01	0.01
Vmax_Brain	96	96	96	96
Km_Brain	18	18	18	18
Vmax_OT	90	90	90	90
Km_OT	180	180	180	180
Vmax_GSIS	0.2	0.32	0.175	0.142857
Km_GSIS	155	193.75	221.42857	310
Km_GSPS	350	437.5	500	700
n_GSPS	4.5	4	4.3	4.2
Vmax_GSPS	0.2	0.32	0.175	0.142857
Vmax_Incretin	5	5	5	5
Km_Incretin	23	23	23	23
Basal_GLP	1	1	1	1
Kd_DPP4	0.1	0.1	0.1	0.1
Kd_aGLP	0.1	0.1	0.1	0.1
Kd_iGLP	0.05	0.05	0.05	0.05
Ka_aGLP	0.00025	0.00025	0.00025	0.00025
bfx	1	0.8	0.7	0.5
afx	1	0.8	0.9	0.8
normal_Vm_GGN	20	20	20	20
normal_BasalGGN	3.25	3.25	3.25	3.25
Km_GGN	0.02	0.02	0.02	0.02
defective_BasalGGN	4	4	4	4
Km_defective_BasalGGN	0.75	0.75	0.75	0.75
K_CPro	20	20	20	20
K_MPro	30	30	30	30

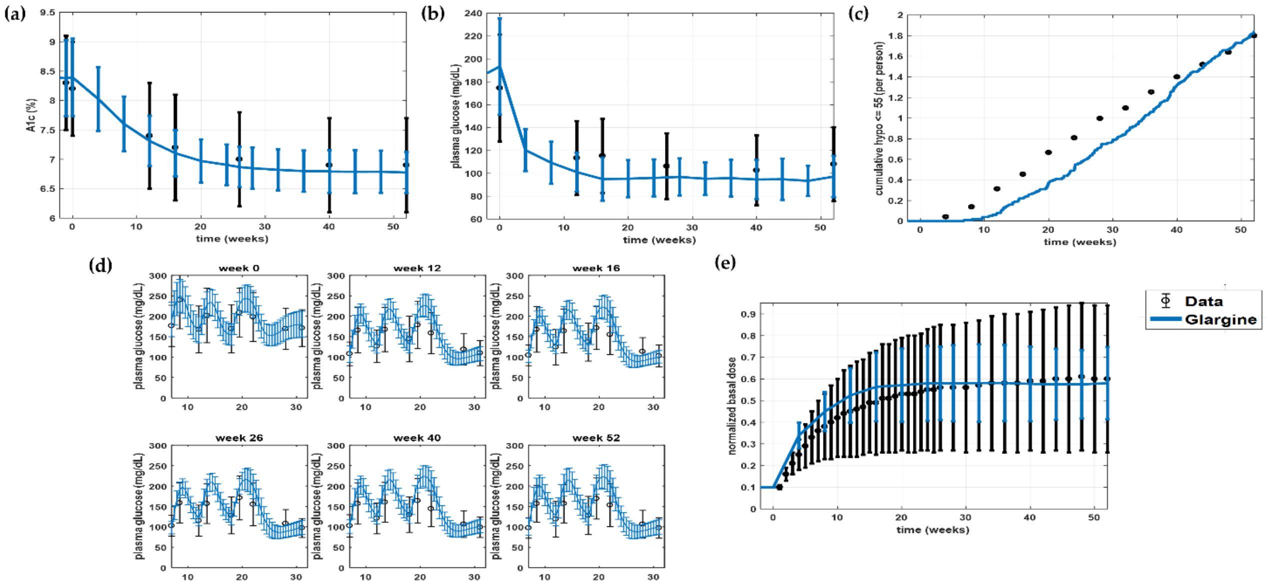

Supplementary Figure S1. Validation of diabetes QSP model with clinical data from Zinman et al (1). (a) plasma glycated hemoglobin over time; (b) fasting plasma glucose concentration over time; (c) cumulative confirmed hypoglycemic episodes; (d) plasma glucose over 32 hours with meals; (e) normalized insulin glargine dose, which is titrated based on the average of prebreakfast blood glucose over 3 days. Blue dots and lines represent mean values of the simulated virtual population, and black, clinical data. Error bars represent standard deviation.

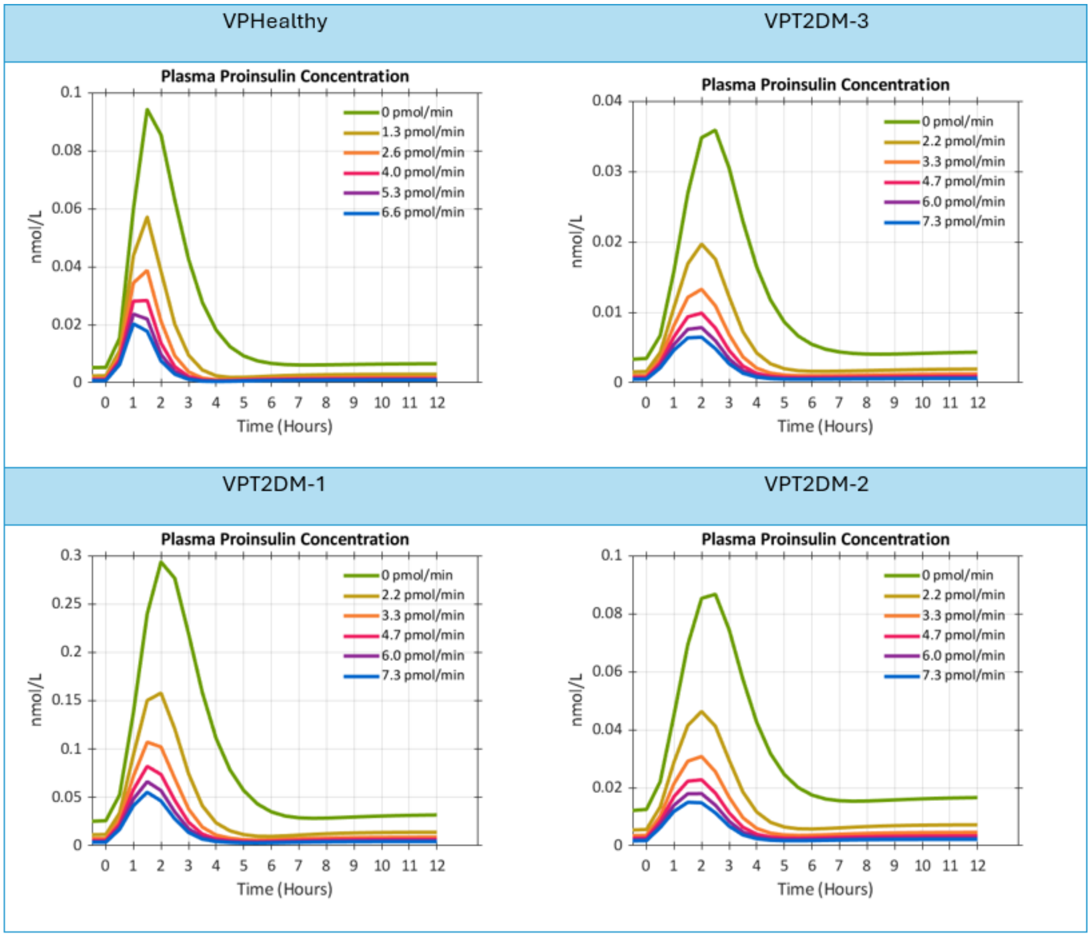

Supplementary Figure S2. Simulated plasma proinsulin concentration assuming varying proinsulin conversion rates during an OGTT in virtual patients.

References

1. Zinman B, Philis-Tsimikas A, Cariou B, Handelsman Y, Rodbard HW, Johansen T, et al. Insulin degludec versus insulin glargine in insulin-naive patients with type 2 diabetes: a 1-year, randomized, treat-to-target trial (BEGIN Once Long). Diabetes Care. 2012;35(12):2464-71.

Supplementary Table S4. IRB approval number and dates Study #11

Trial Number	Site Number	Country	Primary Investigator<br>Site Address	Sub-Investigators	Number of Subjects Enrolled	Name and Address of IEC	Chairperson (if known)	Date of IEC Approvals of Protocol(s) & Amendments*	Description of Incentives
MK-3102-011	0001	United States	Blue, Barney<br>Eminence Research Multispecial<br>6922 S Western Ave, Ste 101<br>Oklahoma City, OK 73139<br>United States	Not applicable	4	Schulman Associates IRB<br>4445 Lake Forest Dr. Ste 300<br>Cincinnati, OH 45242<br>United States	Nelson, Sharon	Prot. 00 – 15-Nov-2012<br>Prot. Am. 02 – 13-May-2013<br>Prot. Am. 03 – 12-Mar-2014	Not applicable
MK-3102-011	0002	United States	Butuk, David<br>Solaris Clinical Research<br>1525 E Leigh Field Dr, #100<br>Meridian, ID 83646<br>United States	Not applicable	10	Schulman Associates IRB<br>4445 Lake Forest Dr. Ste 300<br>Cincinnati, OH 45242<br>United States	Nelson, Sharon	Prot. 00 – 22-Oct-2012<br>Prot. Am. 02 – 02-Jul-2013<br>Prot. Am. 03 – 12-Mar-2014	Not applicable
MK-3102-011	0003	United States	Chuck, Leonard<br>Diablo Clinical Research Inc.<br>2255 Ygnacio Valley Rd, Ste M<br>Walnut Creek, CA 94598<br>United States	Chee, Lambert<br>Christiansen, Mark<br>Stacey, Helen<br>Weinstein, Richard	10	Schulman Associates IRB<br>4445 Lake Forest Dr. Ste 300<br>Cincinnati, OH 45242<br>United States	Nelson, Sharon	Prot. 00 – 19-Oct-2012<br>Prot. Am. 02 – 13-May-2013<br>Prot. Am. 03 – 12-Mar-2014	Not applicable
MK-3102-011	0004	United States	Emmans, Jr., Paul<br>Empirical Clinical Trials, LLC<br>118 S Second St, Ste A<br>Selah, WA 98942<br>United States	Toliver, George	4	Schulman Associates IRB<br>4445 Lake Forest Dr. Ste 300<br>Cincinnati, OH 45242<br>United States	Nelson, Sharon	Prot. 00 – 13-Nov-2012<br>Prot. Am. 02 – 13-May-2013<br>Prot. Am. 03 – 12-Mar-2014	Not applicable
MK-3102-011	0005	United States	Broker, Robert<br>Hillcrest Clinical Research<br>717 SE Main St, Ste B<br>PO Box 1538<br>Simpsonville, SC 29681<br>United States	Ellis, Jennifer<br>Heidt, Francis<br>Johnson, Gretchen<br>Silkiner, David	5	Schulman Associates IRB<br>4445 Lake Forest Dr. Ste 300<br>Cincinnati, OH 45242<br>United States	Nelson, Sharon	Prot. 00 – 15-Nov-2012<br>Prot. Am. 02 – 13-May-2013<br>Prot. Am. 03 – 12-Mar-2014	Not applicable
MK-3102-011	0006	United States	Moran, Joseph<br>Piedmont Healthcare/ Research<br>138 Sherlock Dr<br>Statesville, NC 28625<br>United States	Narendran, Mahendra<br>Nicholson, John	3	Schulman Associates IRB<br>4445 Lake Forest Dr. Ste 300<br>Cincinnati, OH 45242<br>United States	Nelson, Sharon	Prot. 00 – 20-Nov-2012<br>Prot. Am. 02 – 13-May-2013<br>Prot. Am. 03 – 12-Mar-2014	Not applicable
MK-3102-011	0007	United States	Ison, Rodney<br>Community Health Care<br>944 E Cherry St<br>Canal Fulton, OH 83686<br>United States	Not applicable	2	Schulman Associates IRB<br>4445 Lake Forest Dr. Ste 300<br>Cincinnati, OH 45242<br>United States	Nelson, Sharon	Prot. 00 – 12-Nov-2012<br>Prot. Am. 02 – 13-May-2013<br>Prot. Am. 03 – 12-Mar-2014	Not applicable
MK-3102-011	0008	United States	Davidson, Elaine<br>St. Luke's Regional Medical Center<br>215 E Hawaii Ave<br>Nampa, ID 83686<br>United States	Not applicable	1	Schulman Associates IRB<br>4445 Lake Forest Dr. Ste 300<br>Cincinnati, OH 45242<br>United States	Nelson, Sharon	Prot. 00 – 12-Nov-2012<br>Prot. Am. 02 – 13-May-2013<br>Prot. Am. 03 – 12-Mar-2014	Not applicable
MK-3102-011	0009	United States	Williams, John<br>Austin Clinical Research Professionals, LLC<br>18921 NW Second Ave, Ste C<br>Miami, FL 33169<br>United States	Not applicable	1	Schulman Associates IRB<br>4445 Lake Forest Dr. Ste 300<br>Cincinnati, OH 45242<br>United States	Nelson, Sharon	Prot. 00 – 30-Nov-2012<br>Prot. Am. 02 – 13-May-2013<br>Prot. Am. 03 – NAP	Not applicable
MK-3102-011	0010	United States	Weisbrot, Albert<br>Albert J. Weisbrot MD, Inc.<br>7451 S Mason Montgomery Rd, Ste C<br>Mason, OH 45040<br>United States	Not applicable	3	Schulman Associates IRB<br>4445 Lake Forest Dr. Ste 300<br>Cincinnati, OH 45242<br>United States	Nelson, Sharon	Prot. 00 – 29-Nov-2012<br>Prot. Am. 02 – 13-May-2013<br>Prot. Am. 03 – 12-Mar-2014	Not applicable
MK-3102-011	0012	United States	West, James<br>Perimeter North Medical Research<br>1265 Upper Hembree Rd, Ste 200<br>Roswell, GA 30076<br>United States	Not applicable	1	Schulman Associates IRB<br>4445 Lake Forest Dr. Ste 300<br>Cincinnati, OH 45242<br>United States	Nelson, Sharon	Prot. 00 – 03-Dec-2012<br>Prot. Am. 02 – 13-May-2013<br>Prot. Am. 03 – 12-Mar-2014	Not applicable

Trial Number	Site Number	Country	Primary Investigator<br>Site Address	Sub-Investigators	Number of Subjects Enrolled	Name and Address of IEC	Chairperson (if known)	Date of IEC Approvals of Protocol(s) & Amendments*	Description of Incentives
MK-3102-011	0013	United States	Crabtree, Yvette<br>Sunflower Medical Group, P.A.<br>5555 W 58th St.<br>Mission, KS 66202<br>United States	Ragland, Charles<br>Snodell, Thomas	1	Schulman Associates IRB<br>4445 Lake Forest Dr. Ste 300<br>Cincinnati, OH 45242<br>United States	Nelson, Sharon	Prot. 00 – 03-Dec-2012<br>Prot. Am. 02 – 13-May-2013<br>Prot. Am. 03 – 12-Mar-2014	Not applicable
MK-3102-011	0014	United States	Drummond, Waymon<br>Renaissance Clinical Research<br>1151 N Buckner Blvd, Ste 308<br>Dallas, TX 75218<br>United States	Punzi, Henry	4	Schulman Associates IRB<br>4445 Lake Forest Dr. Ste 300<br>Cincinnati, OH 45242<br>United States	Nelson, Sharon	Prot. 00 – 03-Dec-2012<br>Prot. Am. 02 – 13-May-2013<br>Prot. Am. 03 – 12-Mar-2014	Not applicable
MK-3102-011	0015	United States	Ruoff , Gary<br>Westside Family Medical Center<br>6565 W Main St<br>Kalamazoo, MI 49009<br>United States	Boley, Bryon<br>Burhans, Earl<br>Palmitessa, Gabriela	3	Schulman Associates IRB<br>4445 Lake Forest Dr. Ste 300<br>Cincinnati, OH 45242<br>United States	Nelson, Sharon	Prot. 00 – 27-Nov-2012<br>Prot. Am. 02 – 13-May-2013<br>Prot. Am. 03 – 12-Mar-2014	Not applicable
MK-3102-011	0016	United States	Adler, Jay<br>2020 West Colorado Ave, Ste 201-202<br>Colorado Springs, CO 80904<br>United States	Kennedy, Jamieson	1	Schulman Associates IRB<br>4445 Lake Forest Dr. Ste 300<br>Cincinnati, OH 45242<br>United States	Nelson, Sharon	Prot. 00 – 26-Nov-2012<br>Prot. Am. 02 – 13-May-2013<br>Prot. Am. 03 – NAP	Not applicable
MK-3102-011	0017	United States	Lee, Keung<br>Randolph Medical Associates<br>237A N Fayetteville St<br>Asheboro, NC 27203<br>United States	Campbell, Stephen	11	Schulman Associates IRB<br>4445 Lake Forest Dr. Ste 300<br>Cincinnati, OH 45242<br>United States	Nelson, Sharon	Prot. 00 – 29-Nov-2012<br>Prot. Am. 02 – 13-May-2013<br>Prot. Am. 03 – 12-Mar-2014	Not applicable
MK-3102-011	0018	United States	Patel, Rajesh<br>Lycoming Internal Medicine<br>116 Kerr Ave, Ste B<br>Jersey Shore, PA 17740<br>United States	Chapla, Pravinchandra	2	Schulman Associates IRB<br>4445 Lake Forest Dr. Ste 300<br>Cincinnati, OH 45242<br>United States	Nelson, Sharon	Prot. 00 – 07-Dec-2012<br>Prot. Am. 02 – 13-May-2013<br>Prot. Am. 03 – 12-Mar-2014	Not applicable
MK-3102-011	0019	United States	Alwine, Lawrence<br>Brandywine Clinical Research<br>77 Manor Ave, Ste 101<br>Downingtown, PA 19335<br>United States	Kelly, Edward	1	Schulman Associates IRB<br>4445 Lake Forest Dr. Ste 300<br>Cincinnati, OH 45242<br>United States	Nelson, Sharon	Prot. 00 – 19-Nov-2012<br>Prot. Am. 02 – 13-May-2013<br>Prot. Am. 03 – 12-Mar-2014	Not applicable
MK-3102-011	0020	United States	Serje, Jorge<br>NY Total Medical Care, P.C.<br>362 9th St<br>Brooklyn, NY 11215<br>United States	Zevallos, Augusto	2	Schulman Associates IRB<br>4445 Lake Forest Dr. Ste 300<br>Cincinnati, OH 45242<br>United States	Nelson, Sharon	Prot. 00 – 19-Dec-2012<br>Prot. Am. 02 – 13-May-2013<br>Prot. Am. 03 – 12-Mar-2014	Not applicable
MK-3102-011	0022	United States	Huffman, David<br>University Diabetes and Endocrine Consultants<br>5616 Brainerd Rd, Ste 208<br>Chattanooga, TN 37411<br>United States	Not applicable	2	Schulman Associates IRB<br>4445 Lake Forest Dr. Ste 300<br>Cincinnati, OH 45242<br>United States	Nelson, Sharon	Prot. 00 – 14-Mar-2013<br>Prot. Am. 02 – 13-May-2013<br>Prot. Am. 03 – 12-Mar-2014	Not applicable
MK-3102-011	0023	United States	McKenzie, Wilfred<br>M and O Clinical Research, LLC<br>1625 SE 3rd Ave, # 400<br>Fort Lauderdale, FL 33316<br>United States	McKenzie, Rona	1	Schulman Associates IRB<br>4445 Lake Forest Dr. Ste 300<br>Cincinnati, OH 45242<br>United States	Sharon Nelson	Prot. 00 – 15-Apr-2013<br>Prot. Am. 02 – 13-May-2013<br>Prot. Am. 03 – 12-Mar-2014	Not applicable
MK-3102-011	0051	Bulgaria	Klyuchkova, Neli<br>Diagnostic Consultation Center "Equita" EOOD<br>Endocrinology practice<br>5, Tsar Osvoboditel Blvd. and<br>2, Petar Parchevich Str.<br>9000 Varna<br>Bulgaria	Kerekovski, Yuriy	3	Ethics Committee for Multicenter Trial<br>Sveta Nedelya' sqr, No: 5<br>Sofia, 1000<br>Bulgaria	Stoikov, Anastas	Prot. 00 – 17-May- 2013<br>Prot. Am. 02 – 17-Jul-2013<br>Prot. Am. 03 – 21-May-2014	None

Trial Number	Site Number	Country	Primary Investigator<br>Site Address	Sub-Investigators	Number of Subjects Enrolled	Name and Address of IEC	Chairperson (if known)	Date of IEC Approvals of Protocol(s) & Amendments*	Description of Incentives
MK-3102-011	0052	Bulgaria	Ganeva, Silviya<br>"University Multiprofile Hospital for Active Trearment - Dr. Georgi Stranski " EAD<br>Department of Endocrinology and Metabolic Diseases<br>91 and 92, Gen. Vladimir Vazov Str.<br>5800 Pleven<br>Bulgaria	Stoyanova, Beatriche	2	Ethics Committee for Multicenter Trial<br>Sveta Nedelya' sqr, No: 5<br>Sofia, 1000<br>Bulgaria	Stoikov, Anastas	Prot. 00 – NAP<br>Prot. Am. 02 – 17-Jul-2013<br>Prot. Am. 03 – 21-May-2014	None
MK-3102-011	0053	Bulgaria	Marokova, Irinka<br>Diagnostic Consultation Center “Akta Medika” EOOD<br>Endocrinology Practice<br>60, N. Petkov Str.<br>5400 Sevlievo<br>Bulgaria	Dardanov, Hristofor<br>Mandzhukova, Svetoslava<br>Nikolov, Dimitar<br>Peshkov, Ognyan	4	Ethics Committee for Multicenter Trial<br>Sveta Nedelya' sqr, No: 5<br>Sofia, 1000<br>Bulgaria	Stoikov, Anastas	Prot. 00 – 17-May-2013<br>Prot. Am. 02 – 17-Jul-2013<br>Prot. Am. 03 – 21-May-2014	None
MK-3102-011	0054	Bulgaria	Tankova, Tsvetalina<br>University Specialized Hospital for Active Treatment in Endocrinology “Akad. Ivan Penchev” EAD<br>Clinic of Diabetology<br>2, Zdrave Str.<br>1431 Sofia<br>Bulgaria	Chakarova, Nevena	1	Ethics Committee for Multicenter Trial<br>Sveta Nedelya' sqr, No: 5<br>Sofia, 1000<br>Bulgaria	Stoikov, Anastas	Prot. 00 – NAP<br>Prot. Am. 02 – 17-Jul-2013<br>Prot. Am. 03 – 21-May-2014	None
MK-3102-011	0055	Bulgaria	Gushterova, Valentina<br>Multiprofile Hospital for Active Treatment “Puls” AD<br>Endocrinology Practice at Department of Gastroenterology<br>62, Slavyanska Str.<br>2700 Blagoevgrad<br>Bulgaria	Spasova, Vesela	0	Ethics Committee for Multicenter Trial<br>Sveta Nedelya' sqr, No: 5<br>Sofia, 1000<br>Bulgaria	Stoikov, Anastas	Prot. 00 – NAP<br>Prot. Am. 02 – 17-Jul-2013<br>Prot. Am. 03 – 21-May-2014	None
MK-3102-011	0101	Germany	Chevts, Julia<br>Ostendorfstr. 6<br>76199 Karlsruhe, Baden-Württemberg<br>Germany	Reymer, Alla	7	Ethikkommission der Landesärztekammer Hessen<br>Im Vogelsang 3<br>60488 Frankfurt am Main<br>Germany	Harder, Sebastian	Prot. 00 – NAP<br>Prot. Am. 02 – 08-May-2013<br>Prot. Am. 03 – 27-Mar-2014	None
MK-3102-011	0102	Germany	Niemetz, Ingo<br>Diabetologische Schwerpunktpra<br>Kurfürstenstraße 10,<br>Kassel, Hessen, 34117<br>Germany	Pollmächer, Stefan	0	Ethikkommission der Landesärztekammer Hessen<br>Im Vogelsang 3<br>60488 Frankfurt am Main<br>Germany	Harder, Sebastian	Prot. 00 – NAP<br>Prot. Am. 02 – 08-May-2013<br>Prot. Am. 03 – 27-Mar-2014	None
MK-3102-011	0103	Germany	Ott, Petra<br>Rabenauer Strasse 9<br>Dippoldiswalde 01744 Sachsen<br>Germany	Hoffmann, Caren	0	Ethikkommission der Landesärztekammer Hessen<br>Im Vogelsang 3<br>60488 Frankfurt am Main<br>Germany	Harder, Sebastian	Prot. 00 – NAP<br>Prot. Am. 02 – 08-May-2013<br>Prot. Am. 03 – 27-Mar-2014	None
MK-3102-011	0104	Germany	Thinesse-Mallwitz, Manuela<br>Faeustlestr. 3<br>80339 München, Bayern<br>Germany	Hartard, Diana	8	Ethikkommission der Landesärztekammer Hessen<br>Im Vogelsang 3<br>60488 Frankfurt am Main<br>Germany	Harder, Sebastian	Prot. 00 – NAP<br>Prot. Am. 02 – 08-May-2013<br>Prot. Am. 03 – 27-Mar-2014	None
MK-3102-011	0105	Germany	Remppis, Rudolf<br>Bahnhofstr. 14 Weißenhorn<br>89264 Bayern<br>Germany	Röth, Elisabeth	0	Ethikkommission der Landesärztekammer Hessen<br>Im Vogelsang 3<br>60488 Frankfurt am Main<br>Germany	Harder, Sebastian	Prot. 00 – NAP<br>Prot. Am. 02 – 08-May-2013<br>Prot. Am. 03 – 27-Mar-2014	None
MK-3102-011	0106	Germany	Khariouzov, Andrei<br>Robert-Rössle-Str. 10 Haus 85 D<br>13125 Berlin<br>Germany	Djacenko, Svetlana<br>Lieb, Christian	6	Ethikkommission der Landesärztekammer Hessen<br>Im Vogelsang 3<br>60488 Frankfurt am Main<br>Germany	Harder, Sebastian	Prot. 00 – NAP<br>Prot. Am. 02 – 08-May-2013<br>Prot. Am. 03 – 27-Mar-2014	None

Trial Number	Site Number	Country	Primary Investigator<br>Site Address	Sub-Investigators	Number of Subjects Enrolled	Name and Address of IEC	Chairperson (if known)	Date of IEC Approvals of Protocol(s) & Amendments*	Description of Incentives
MK-3102-011	0108	Germany	Peldschus, Meike<br>Klinische Forschung Berlin-Mitte GmbH Georgenstrasse 24<br>20253 Hamburg<br>Germany	Hahn, Michael<br>Lieb, Christian<br>Mumme, Angelina<br>Restemeyer, Cordula<br>Thron, Andrea	4	Ethikkommission der Landesärztekammer Hessen<br>Im Vogelsang 3<br>60488 Frankfurt am Main<br>Germany	Harder, Sebastian	Prot. 00 – NAP<br>Prot. Am. 02 – 08-May-2013<br>Prot. Am. 03 – 27-Mar-2014	None
MK-3102-011	0109	Germany	Nischik, Ruth<br>Friedrich-Ebert-Str. 33<br>04109 Leipzig, Sachsen<br>Germany	Schubert, Kristin	2	Ethikkommission der Landesärztekammer Hessen<br>Im Vogelsang 3<br>60488 Frankfurt am Main<br>Germany	Harder, Sebastian	Prot. 00 – NAP<br>Prot. Am. 02 – 08-May-2013<br>Prot. Am. 03 – 27-Mar-2014	None
MK-3102-011	0110	Germany	Kast, Petra<br>Lohmühlenstr. 5 Haus O<br>20099 Hamburg<br>Germany	Bergmann, Regine<br>Schmitt, Susanne	1	Ethikkommission der Landesärztekammer Hessen<br>Im Vogelsang 3<br>60488 Frankfurt am Main<br>Germany	Harder, Sebastian	Prot. 00 – NAP<br>Prot. Am. 02 – 08-May-2013<br>Prot. Am. 03 – 27-Mar-2014	None
MK-3102-011	0111	Germany	Heymer, Peter<br>Prager Strasse 10 Sachsen<br>01069 Dresden<br>Germany	Günzel, Andrea<br>Winkler, Jan	5	Ethikkommission der Landesärztekammer Hessen<br>Im Vogelsang 3<br>60488 Frankfurt am Main<br>Germany	Harder, Sebastian	Prot. 00 – NAP<br>Prot. Am. 02 – 08-May-2013<br>Prot. Am. 03 – 27-Mar-2014	None
MK-3102-011	0112	Germany	Stratmann, Liebhild<br>Georgenstrasse 24<br>10117 Berlin<br>Germany	Dietzel, Joanna<br>Djacenko, Svetlana<br>von Muenchhausen, Candy	3	Ethikkommission der Landesärztekammer Hessen<br>Im Vogelsang 3<br>60488 Frankfurt am Main<br>Germany	Harder, Sebastian	Prot. 00 – NAP<br>Prot. Am. 02 – 08-May-2013<br>Prot. Am. 03 – 27-Mar-2014	None
MK-3102-011	0151	Hungary	Vadász, János<br>Hetényi Géza Kórház<br>H-5004 Szolnok<br>Tószeg u. 21<br>Hungary	Barta, György	6	Jász-Nagykun-Szolnok Megyei Hetényi Géza Kórház-Rendelőintézet Intézeti Kutatásetikai Bizottsága<br>H-5004 Szolnok<br>Tószeg u. 21<br>Hungary	Bakos, Noémi	Prot. 00 – 25-Jan-2013<br>Prot. Am. 02 – 10-Jun-2013<br>Prot. Am. 03 – 03-Jun-2014	None
MK-3102-011	0152	Hungary	Taller, András<br>Uzsoki utcai Kórház<br>H-1145 Budapest<br>Uzsoki u. 39-45<br>Hungary	Kovács, István<br>Szántó, Péter	2	Uzsoki utcai Kórház Helyi Etikai Bizottsága<br>H-1145 Budapest<br>Uzsoki u. 29-41<br>Hungary	Milibák, Tibor	Prot. 00 – 25-Jan-2013<br>Prot. Am. 02 – 10-Jun-2013<br>Prot. Am. 03 – 03-Jun-2014	None
MK-3102-011	0153	Hungary	Salamon, Csaba<br>Clinfan Kft.<br>H-7100 Szekszárd<br>Pollack Mihály u. 50<br>Hungary	Németh, Csaba<br>Sudár, Zsolt	10	Tolna Megyei Balassa János Kórház, Kórházi Etikai Bizottság<br>H-7100 Szekszárd<br>Béri Balogh Ádám utca 5-7<br>Hungary	Vastag, Oszkár	Prot. 00 – 25-Jan-2013<br>Prot. Am. 02 – 10-Jun-2013<br>Prot. Am. 03 – 03-Jun-2014	None
MK-3102-011	0154	Hungary	Kónyi, Attila<br>COROMED SMO Kft.<br>H-7623 Pécs<br>Jászai Mari utca 3<br>Hungary	Gaszner, Balázs<br>Magyari, Balázs<br>Pintér, István<br>Sárszegi, Zsolt	3	Pécsi Tudományegyetem Klinikai Központ Intézményi és Dél-dunántúli Regionális Kutatásetikai Bizottsága<br>H-7623 Pécs<br>Rákóczi út 2<br>Hungary	Kosztolányi, György	Prot. 00 – 25-Jan-2013<br>Prot. Am. 02 – 10-Jun-2013<br>Prot. Am. 03 – 03-Jun-2014	None
MK-3102-011	0155	Hungary	Faludi, Péter<br>XV. Kerületi Önkormányzat Egészségügyi Intézménye<br>H-1158 Budapest<br>Őrjárat u. 1-5<br>Hungary	Bende, Ilona<br>Sebestyén, Júlia	3	Uzsoki utcai Kórház Helyi Etikai Bizottsága<br>H-1145 Budapest<br>Uzsoki u. 29-41<br>Hungary	Milibák, Tibor	Prot. 00 – 25-Jan-2013<br>Prot. Am. 02 – 10-Jun-2013<br>Prot. Am. 03 – 03-Jun-2014	None
MK-3102-011	0156	Hungary	Fövényi, József<br>Péterfy Sándor utcai Kórház<br>H-1076 Budapest<br>Péterfy Sándor u. 8-20<br>Hungary	Lehotkai, Lajos<br>Sallai, Tamás<br>Thaisz, Erzsébet	3	Péterfy Sándor Utcai Kórház-Rendelőintézet és Baleseti Központ Intézményi Kutatásetikai Bizottsága<br>H-1076 Budapest<br>Péterfy Sándor u. 8-20<br>Hungary	Vas, Mária	Prot. 00 – 25-Jan-2013<br>Prot. Am. 02 – 10-Jun-2013<br>Prot. Am. 03 – 03-Jun-2014	None

Trial Number	Site Number	Country	Primary Investigator<br>Site Address	Sub-Investigators	Number of Subjects Enrolled	Name and Address of IEC	Chairperson (if known)	Date of IEC Approvals of Protocol(s) & Amendments*	Description of Incentives
MK-3102-011	0157	Hungary	Forster, Tamás<br>Szegedi Tudományegyetem Szent-Györgyi Albert Klinikai Központ, Általános Orvostudományi Kar<br>H-6720 Szeged<br>Korányi fasor 6<br>Hungary	Farkas, Attila<br>Jebelovszki, Éva	3	Szegedi Tudományegyetem Szent-Györgyi Albert Klinikai Központ, Humán Orvosbiológiai Intézményi és Regionális Kutatásetikai Bizottsága<br>H-6720 Szeged<br>Korányi fasor 8-10<br>Hungary	Wittmann, Tibor	Prot. 00 – 25-Jan-2013<br>Prot. Am. 02 – 10-Jun-2013<br>Prot. Am. 03 – 03-Jun-2014	None
MK-3102-011	0158	Hungary	Kiss, Krisztián<br>Meditoll Kft.<br>H-2100 Gödöllő<br>Arany János u. 20<br>Hungary	Bakonyi, Géza<br>Bandúr, Szilvia<br>Koncz, Judit	9	Pest Megyei Flór Ferenc Kórház , Helyi Etikai Bizottság<br>H-2143 Kistarcsa<br>Semmelweis tér 1<br>Hungary	Simonyi, Gábor	Prot. 00 – 25-Jan-2013<br>Prot. Am. 02 – 10-Jun-2013<br>Prot. Am. 03 – 03-Jun-2014	None
MK-3102-011	0159	Hungary	Piros, Györgyike<br>Szegedi Tudományegyetem Szent-Györgyi Albert Klinikai Központ<br>Általános Orvostudományi Kar<br>H-6725 Szeged<br>Semmelweis u 6/B<br>Hungary	Kiszely, Ildikó<br>Forster, Tamás	5	Szegedi Tudományegyetem Szent-Györgyi Albert Klinikai Központ, Humán Orvosbiológiai Intézményi és Regionális Kutatásetikai Bizottsága<br>H-6720 Szeged<br>Korányi fasor 8-10<br>Hungary	Wittmann, Tibor	Prot. 00 – 25-Jan-2013<br>Prot. Am. 02 – 10-Jun-2013<br>Prot. Am. 03 – 03-Jun-2014	None
MK-3102-011	0251	Italy	Borghi, Claudio<br>Via Pietro Albertoni 15<br>40138 Bologna<br>Italy	Cicero, Arrigo F.G.<br>Veronesi, Maddalena<br>Rosticci, Martina	0	Comitato Etico Indipendente<br>Azienda Ospedaliero-Universitaria Policlinico S. Orsola-Malpighi di Bologna<br>Via Albertoni, 15<br>40138 Bologna<br>Italy	Unknown	Prot. 00 – 15-Mar-2013<br>Prot. Am. 02 – 10-Sep-2013<br>Prot. Am. 03 - NAP	None
MK-3102-011	0254	Italy	Gambardella, Sergio<br>Universita Tor Vergata Asl Rom<br>Via Duilio Cambellotti, 11<br>Roma 00133<br>Italy	Santini, Valina	0	Unknown	Unknown	Unknown	None
MK-3102-011	0255	Italy	Di Carlo, Alberto Michele<br>Azienda USL n. 2 Lucca Ospedal<br>Via dell'Ospedale<br>Lucca 55100<br>Italy	Casadidio, Ilaria	0	Unknown	Unknown	Unknown	None
MK-3102-011	0256	Italy	Derosa, Giuseppe<br>Piazzale Golgi 19<br>27100 Pavia<br>Italy	Not applicable	6	Comitato di Bioetica<br>Fondazione IRCCS Policlinico San Matteo<br>Direzione Scientifica (Padiglione 10B) Piazzale Golgi, 19<br>27100 Pavia<br>Italy	Unknown	Prot. 00 – 17-Dec-2012<br>Prot. Am. 02 – 03-Jun-2013<br>Prot. Am. 03 – 05-May-2014	None
MK-3102-011	0257	Italy	Buzzetti, Rafaella<br>Via Antonio Canova<br>04100 Latina<br>Italy	Foffi, Chiara<br>Leto, Gaetano<br>Moretti, Chiara	1	Comitato Etico<br>ASL RM/C di Roma<br>Via Primo Carnera, 1<br>00142 Roma<br>Italy	Unknown	Prot. 00 – 17-Jan-2013<br>Prot. Am. 02 – 04-Jul-2013<br>Prot. Am. 03 – 24-Jun-2014	None
MK-3102-011	0258	Italy	Boemi, Massimo<br>Via della Montagnola 81<br>60125 Ancona<br>Italy	Brandoni, Gabriele<br>D'Angelo, Federica<br>Rabini, Rosa Anna	6	Comitato di Bioetica<br>I.N.R.C.A. – Istituto Nazionale di Ricovero e Cura per Anziani<br>Via della Monagnola 81<br>60125 ANCONA<br>Italy	Unknown	Prot. 00 – 24-Jan-2013<br>Prot. Am. 02 – 20-Jun-2013<br>Prot. Am. 03 – 12-Jun-2014	None
MK-3102-011	0301	Korea	Park, Joong-Yeol<br>88, Olympic-ro 43-gil<br>Sonpa-gu, 138-736 Seoul Teugbyeolsi<br>Korea	Jang, Jung Eun<br>Jung, Chang Hee<br>Leem, Jaechan	5	Asan Medical center Institutional Review Bord<br>88, Olympic-ro 43-gil<br>Sonpa-gu, Seoul, 138-736<br>Korea	Chung, Jong-Woo	Prot. 00 – 06-May-2013<br>Prot. Am. 02 – 14-Jun-2013<br>Prot. Am. 03 – 23-May-2014	None

Trial Number	Site Number	Country	Primary Investigator<br>Site Address	Sub-Investigators	Number of Subjects Enrolled	Name and Address of IEC	Chairperson (if known)	Date of IEC Approvals of Protocol(s) & Amendments*	Description of Incentives
MK-3102-011	0302	Korea	Yoon, Kun Ho<br>222 Banpo-daero<br>Seocho-gu Seoul 137-701<br>Seoul Teugbyeolsi [Seoul-T'ukp]<br>Korea	Yang, Hae Kyung<br>Kim, Hun-Sung	1	Seoul St. Mary's Hospital Institutional Review Bord<br>222 Banpo-daero<br>Seocho-gu, Seoul, 137-701<br>Korea	Unknown	Prot. 00 – 20-May-2013<br>Prot. Am. 02 – 24-Jun-2013<br>Prot. Am. 03 – 17-Jun-2014	None
MK-3102-011	0303	Korea	Park, Ie Byung<br>Gachon University Gil Medical Center<br>21, Namdong-daero 774<br>Namdong-gu, Incheon, 405-760<br>Korea	Eom, Young Sil	1	Gachon University Gil Medical Center,<br>21, Namdong-daero 774<br>Namdong-gu, Incheon, 405-760<br>Korea	Lee, Gun	Prot. 00 – 24-Jan-2013<br>Prot. Am. 02 – 25-Jun-2013<br>Prot. Am. 03 ¬ 05-Nov-2013	None
MK-3102-011	0304	Korea	Park, Seokwon<br>CHA Bundang Medical Center, CHA University<br>Yatap-ro 59<br>Bundang-Gu Seongnam-si Gyeonggi-do 463-712<br>Korea	Cho, Yong-Wook<br>Kim, Kyung Soo<br>Kim, Soo-Kyung	3	CHA Bundang Medical Center, CHA University<br>Yatap-ro 59<br>Bundang-Gu Seongnam-si Gyeonggi-do, 487-010<br>Korea	Chi, Hoon Sang	Prot. 00 – 18-Mar-2013<br>Prot. Am. 02 – 03-Jul-2013<br>Prot. Am. 03 – 07-May-2014	None
MK-3102-011	0305	Korea	Lee, Moon-Kyu<br>81, Irwon-ro, Gangnam-gu<br>135-710 Seoul Teugbyeolsi [Seoul-T'ukp] 135-710 Seoul<br>Korea	Jin, Sang-Man<br>Oh, Sewon<br>Park, Sun Mi	1	Samsung Medical center Institutional Review Bord<br>81, Irwon-ro<br>Gangnam-gu, Seoul, 135-710<br>Korea	Unknown	Prot. 00 – 10-May-2013<br>Prot. Am. 02 – 21-Jun-2013<br>Prot. Am. 03 – 08-May-2014	None
MK-3102-011	0306	Korea	Sung, Yeon Ah<br>1071, Anyangcheon-ro, Yangcheon-Ku<br>158-710 Seoul Teugbyeolsi [Seoul-T'ukp] 158-710 Seoul<br>Korea	Lee, Hye Jin<br>Oh, Jee Young<br>Song, Do Kyeong	1	Ewha Womans University Mokdong Hospital Institutional Review Bord<br>1071, Anyangcheon-ro,<br>YangCheon-Ku, Seoul, 158-710<br>Korea	Pyun, Wook-Bum	Prot. 00 – 20-Mar-2013<br>Prot. Am. 02 – 05-Jun-2013<br>Prot. Am. 03 – 07-May-2014	None
MK-3102-011	0351	Netherlands	Mevissen, Harry<br>Nijveenlaan 25 Wildervank<br>9648 BE Groningen<br>Netherlands	Feis, Wijnand	15	Commissie Medische Ethiek LUMC<br>Albinusdreef 2, Postbus 9600<br>2300 RC Leiden<br>Netherlands	Unknown	Prot. 00 - 16-May-2013<br>Prot. Am. 02 – 26-Nov-13<br>Prot. Am 03 – 12-May-2014	None
MK-3102-011	0352	Netherlands	Pijl, Hanno<br>Leids Universitair Medisch Centrum (LUMC)<br>P.O. Box 9600<br>Leiden 2300 RC<br>Netherlands	Jazet, Ingrid	0	Commissie Medische Ethiek LUMC<br>Albinusdreef 2, Postbus 9600<br>2300 RC Leiden<br>Netherlands	Unknown	Prot. 00 - 16-May-2013<br>Prot. Am. 02 – 26-Nov-13<br>Prot. Am 03 – 12-May-2014	None
MK-3102-011	0353	Netherlands	van de Walle, Vivienne<br>Elsstraat 47 Beek<br>6191JW Limburg<br>Netherlands	Not applicable	2	Commissie Medische Ethiek LUMC<br>Albinusdreef 2, Postbus 9600<br>2300 RC Leiden<br>Netherlands	Unknown	Prot. 00 – 16-May-2013<br>Prot. Am. 02 – 26-Nov-2013<br>Prot. Am. 03 – 12-May-2014	None
MK-3102-011	0401	Philippines	Gomez, Olivert<br>Docbebet Diabetes Clinic<br>Pau Street, Brgy. Calulut<br>San Fernando, Pampanga 2000<br>Philippines	Payumo, Elaine	7	Ethics Committee<br>Mother Theresa of Calcutta Medical Center<br>Barangay Maimpis<br>San Fernando, Pampanga 2000<br>Philippines	Panganiban, Ronaldo M.	Prot. 00 – 22-Mar-2013<br>Prot. Am. 02 – 20-Jan-2014<br>Prot. Am. 03 – 27-May-201	None
MK-3102-011	0402	Philippines	Sison, Jorge<br>Medical Center Manila<br>Room 24 Mezzanine<br>1122 General Luna St<br>Ermita, Manila 1000<br>Philippines	Manua, Norhanah<br>Vieja, Dianne	4	ManilaMed Ethics Review Committee<br>Medical Center Manila<br>U.N. Avenue cor. Taft Avenue<br>Ermita, Manila 1000<br>Philippines	De Jesus, Virgina R	Prot. 00 – 04-Jul-2013<br>Prot. Am. 2 – 11-Nov-2013<br>Prot. Am. 3 – 14-Aug-2014	None
MK-3102-011	0403	Philippines	Morales-Palomares, Ellen<br>Private Clinic of Dr. Ellen Morales – Palomares, C.A.,<br>Samonte Street, Barangay 5<br>Laoag City, Ilocos Norte, 2900<br>Philippines	Manuel, Jovie Joy	5	Research Ethics Review Committee<br>Mariano Marcos Memorial Hospital and Medical Center<br>Batac City, Ilocos Norte 2906<br>Philippines	Abadilla-Felizar, Marcia	Prot. 00 – 19-Aug-2013<br>Prot. Am. 2 – 15-May-2014<br>Prot. Am. 3 – 15-May-2014	None

Trial Number	Site Number	Country	Primary Investigator<br>Site Address	Sub-Investigators	Number of Subjects Enrolled	Name and Address of IEC	Chairperson (if known)	Date of IEC Approvals of Protocol(s) & Amendments*	Description of Incentives
MK-3102-011	0404	Philippines	Romero, Chela Marie<br>Cebu Doctors' University Hospital<br>Room 203-A Medical Arts Building 1<br>Osmeña Boulevard<br>Cebu City 6000<br>Philippines	Bitoon, Wynrose	10	Research Ethics Committee<br>Cebu Doctors' University Hospital<br>Ground Floor, Administrative Building<br>Gov. M. Roa St. cor. Don Jose Avila St<br>Cebu City 6000<br>Philippines	Alsay-Uy, Ma. Noemi	Prot. 00 – 02-May-2013<br>Prot. Am. 2 – 23-Jul-2013<br>Prot. Am. 3 – 09-Jun-2014	None
MK-3102-011	0405	Philippines	Tan, Iris Thiele<br>University of the Philippines – Philippine General Hospital<br>Endocrinology Office, Medical Research Laboratory<br>Taft Avenue<br>Ermita, Manila 1000<br>Philippines	Urbanozo, Hannah	3	University of the Philippines Manila Research Ethics Board<br>UP Manila<br>2nd Floor Paz Mendoza Building<br>College of Medicine<br>547 Pedro Gil Street<br>Ermita, Manila 1000<br>Philippines	Mantaring III, Jacinto Blas V.	Prot. 00 – 07-Aug-2013<br>Prot. Am. 2 – 14-Nov-2013<br>Prot. Am. 3 – 27-Aug-2014	None
MK-3102-011	0406	Philippines	Verdillo, Leah Nita<br>Siliman University Medical Center Foundation, Inc.<br>Preventive and Community Medicine Department<br>Aldecoa Road<br>Dumaguete City 6200<br>Philippines	Ursos, Ma. Lourdes	7	Research Committee<br>Silliman University Medical Center Foundation, Inc.<br>Dumaguete City 6200<br>Philippines	Ursos, Walden R.	Prot. 00 – 10-Jun-2013<br>Prot. Am. 2 – 15-Aug-2013<br>Prot. Am. 3 – 20-Jun- 2014	None
MK-3102-011	0451	Romania	Ferariu, Ioana Emilia<br>Diabol SRL<br>Str.Calea Bucuresti nr.62<br>Bloc A13, sc. A, ap.3<br>Brasov, 500365<br>Romania	Zarnescu, Mihaela Liliana	10	CEC: Comisia Naţională de Bioetică a Medicamentului şi a Dispozitivelor Medicale<br>Sos. Stefan cel Mare nr.19-21 sect.2<br>Bucuresti, 020125<br>Romania	Antonescu, Dinu	Prot. 00– 08-May-2013<br>Prot. Am. 02 – 16-Jul-2013<br>Prot. Am. 03 – 22-May-2014	None
MK-3102-011	0452	Romania	Romanescu, Dana Diana<br>Pelican Impex SRL<br>Str. Corneliu Coposu nr.2<br>Oradea, 410469<br>Romania	Calin, Cristina Anca	13	CEC: Comisia Naţională de Bioetică a Medicamentului şi a Dispozitivelor Medicale<br>Sos. Stefan cel Mare nr.19-21 sect.2<br>Bucuresti, 020125<br>Romania	Antonescu, Dinu	Prot. 00 – 08-May-2013<br>Prot. Am. 02 – 16-Jul-2013<br>Prot. Am. 03 – 22-May-2014	None
MK-3102-011	0453	Romania	Vacaru, Georgeta<br>Easydiet SRL<br>Str. Grigore Alexandrescu nr.80<br>Bucuresti, 010627<br>Romania	Coravu, Diana	2	CEC: Comisia Naţională de Bioetică a Medicamentului şi a Dispozitivelor Medicale<br>Sos. Stefan cel Mare nr.19-21 sect.2<br>Bucuresti, 020125<br>Romania	Antonescu, Dinu	Prot. 00 – 08-May-2013<br>Prot. Am. 02 – 16-Jul-2013<br>Prot. Am. 03 – 22-May-2014	None
MK-3102-011	0454	Romania	Radulian, Gabriela<br>Institutul National de Diabet, Nutritie si Boli Metabolice "N.C.Paulescu"<br>Sectia Clinica II Diabet, nutritie si boli metabolice<br>Str. Ion Movila nr.5-7<br>Bucuresti, 020475<br>Romania	Nan, Raluca	3	CEC: Comisia Naţională de Bioetică a Medicamentului şi a Dispozitivelor Medicale<br>Sos. Stefan cel Mare nr.19-21 sect.2<br>Bucuresti, 020125<br>Romania	Antonescu, Dinu	Prot. 00 – 08-May-2013<br>Prot. Am. 02 – 16-Jul-2013<br>Prot. Am. 03 – 22-May-2014	None
MK-3102-011	0455	Romania	Halmagyi, Ildiko Stefania<br>Centrul Medical de Diagnostic si Tratament Ambulator Neomed SRL<br>Str.Crisului nr.1<br>Bloc 1, sc. C, ap. 2<br>Brasov, 500283<br>Romania	Bancu, Mihaela<br>Cofaru, Brandusa<br>Neculicioiu, Cristina	15	CEC: Comisia Naţională de Bioetică a Medicamentului şi a Dispozitivelor Medicale<br>Sos. Stefan cel Mare nr.19-21 sect.2<br>Bucuresti, 020125<br>Romania	Antonescu, Dinu	Prot. 00 – 08-May-2013<br>Prot. Am. 02 – 16-Jul-2013<br>Prot. Am. 03 – 22-May-2014	None
MK-3102-011	0456	Romania	Graur, Mariana<br>Spitalul Clinic Judetean de Urgente "Sf. Spiridon" Iasi<br>Diabet zaharat, nutritie si boli metabolice, Bld. Independentei nr.1<br>Iasi, 700111<br>Romania	Mihalache, Laura	0	CEC: Comisia Naţională de Bioetică a Medicamentului şi a Dispozitivelor Medicale<br>Sos. Stefan cel Mare nr.19-21 sect.2<br>Bucuresti, 020125<br>Romania	Antonescu, Dinu	Prot. 00 – 08-May-2013<br>Prot. Am. 02 – 16-Jul-2013<br>Prot. Am. 03 – 22-May-2014	None

Trial Number	Site Number	Country	Primary Investigator<br>Site Address	Sub-Investigators	Number of Subjects Enrolled	Name and Address of IEC	Chairperson (if known)	Date of IEC Approvals of Protocol(s) & Amendments*	Description of Incentives
MK-3102-011	0457	Romania	Morosanu, Magdalena<br>Cabinet Medical Individual Morosanu V.Magdalena<br>Str.Constructorilor nr.25<br>bloc E5, scara 4, ap.64<br>Galati, 800371<br>Romania	Morosanu, Andreea	6	CEC: Comisia Naţională de Bioetică a Medicamentului şi a Dispozitivelor Medicale<br>Sos. Stefan cel Mare nr.19-21 sect.2<br>Bucuresti, 020125<br>Romania	Antonescu, Dinu	Prot. 00 – 08-May-2013<br>Prot. Am. 02 – 16-Jul-2013<br>Prot. Am. 03 – 22-May-2014	None
MK-3102-011	0501	Taiwan	Pei, Dee<br>Cardinal Tien Hospital<br>Room 818, 8th Floor<br>Building A, No.362<br>Jhongjheng Rd, Xindian Dist<br>New Taipei City 23148<br>Taiwan	Chung, Ming-Min<br>Hsia, Te-Lin<br>Ma, Wen-Ya<br>Su, Ching-Chieh	3	Institutional Review Board, Cardinal Tien Hospital<br>Room 821, 8th Floor<br>Building A, No.362<br>Jhongjheng Rd, Xindian Dist<br>New Taipei City 23148<br>Taiwan	Chou, Ting-Ywan	Prot. 00 – 02-Jul-2013<br>Prot. Am. 02 – 30-Sep-2013<br>Prot. Am. 03 – 08-Jul-2014	None
MK-3102-011	0502	Taiwan	Yen, Feng-Chieh<br>Chi Mei Medical Center<br>2F, Department of Endocrinology<br>No.901,Chung-Hwa road<br>Yongkang City, Tainan City 71004<br>Taiwan	Chou, Chien-Wen<br>Tien, Kai-Jen<br>Yang, Chwen-Yi	1	Institutional Review Board<br>Chi Mei Medical Center, Tainan, Taiwan<br>4th Floor, 3rd Medical Building<br>No.901,Chung-Hwa road<br>Yongkang City, Tainan City 71004<br>Taiwan	Tsai, Yung-Chieh	Prot. 00 – 14-Mar-2013<br>Prot. Am. 02 – 12-Sep-2013<br>Prot. Am. 03 – 12-Jun-2014	None
MK-3102-011	0503	Taiwan	Chang, Chih-Jen<br>Traditional Chinese Medicine Clinical Center<br>5th Floor, Outpatient Clinic Building, No.138<br>Sheng Li Road, Tainan City 704<br>Taiwan	Wu, Jin-Shang<br>Yang, Yi-Ching	9	Institutional Review Board<br>National Cheng Kung University Hospital<br>138 Sheng-Li Rd<br>Tainan 704<br>Taiwan R.O.C	Lin, Thy-Sheng	Prot. 00 –22-Apr-2013<br>Prot. Am. 02 –31-Jul-2013<br>Prot. Am. 03 – 09-Jun-2014	None
MK-3102-011	0505	Taiwan	Chuang, Lee-Ming<br>National Taiwan University Hospital<br>Rm 5418, 4F, 5 East, No.1 Changde St<br>Taipei City 100, (R.O.C.)<br>Taiwan	Chang, Tien-Jyun<br>Jiang, Yi-Der<br>Li, Hung-Yuan<br>Lu, Jin-Ying	3	Research Ethics Committee,<br>2F, West Site Original Building, No.1 Changde St<br>Taipei City 100, (R.O.C.)<br>Taiwan	Ho, Hong-Nerng	Prot. 00 – 19-Feb-2013<br>Prot. Am. 02 – 19-Aug-2013<br>Prot. Am. 03 – 18-Jun-2014	None
MK-3102-011	0507	Taiwan	Chang, Chwen-Tzuei<br>China Medical University Hospital<br>Department of Internal Medicine<br>B1 Li-Fu Medical Building<br>2 Yude Road<br>Taichung, 40447, (R.O.C.)<br>Taiwan	Chen, Ching-Chu	11	Research Ethics Committee<br>9F, First Medical Building<br>No. 2 Yude Road<br>Taichung, 40447, (R.O.C.)<br>Taiwan	Fuh, Mao-Tsu	Prot. 00 – 08-May-2013<br>Prot. Am. 02 – 28-Aug-2013<br>Prot. Am. 03 – 04-Jun-2014	None
MK-3102-011	0508	Taiwan	Lin, Ching-Ling<br>Cathay General Hospital<br>Patient Education Office next to Outpatient Room 17<br>1st Floor, Main Building<br>280 Ren-Ai Road. Sec.4<br>Taipei 106<br>Taiwan	Huang, Li-Chi	1	Institutional Review Board of the Cathay General Hospital<br>No. 280, Sec. 4 Ren Ai Road<br>Taipei 10630, (R.O.C)<br>Taiwan	Huang, Cheng-Hua	Prot. 00 – 10-Jul-2013<br>Prot. Am. 02 – 29-Oct-2013<br>Prot. Am. 03 – 18-Jun-2014	None
MK-3102-011	0509	Taiwan	Hsieh, An-Tsz<br>Taipei Medical University- Sha<br>Clinical Research Center<br>2nd Floor, Administrative Building<br>No.291, Jhongjheng Rd<br>Jhonghe City, Taipei 235<br>Taiwan	Lin, Jiunn-Diann<br>Wu, Chung-Ze	1	TMU-Joint Institutional Review Board<br>250, Wu Hsing Street<br>Taipei 11031<br>Taiwan	Hsueh, Jui-Yuan	Prot. 00 – 14- May-2013<br>Prot. Am. 02 – 03-Sep-2013<br>Prot. Am. 03 – 02-Jul-2014	None
MK-3102-011	0511	Taiwan	Lu, Yung Chuan<br>E-Da Hospital<br>Department of Internal Medicine<br>6F, Building C<br>No.1, Yida Road<br>Jiaosu Village, Yanchao District<br>Kaohsiung City 82445, (R.O.C.)<br>Taiwan	Huang, Ju-Chun	4	Institutional Review Board<br>No.6, Yida Road<br>Jiaosu Village, Yanchao District<br>Kaohsiung City 82445, (R.O.C.)<br>Taiwan	Lin, His-Hsun	Prot. 00 – 08-Apr-2013<br>Prot. Am. 02 – 12-Sep-2013<br>Prot. Am. 03 – 03-Jul-2014	None

**Trial Number**	**Site Number**	**Country**	**Primary Investigator**<br>**Site Address**	**Sub-Investigators**	**Number of Subjects Enrolled**	**Name and Address of IEC**	**Chairperson (if known)**	**Date of IEC Approvals of Protocol(s) & Amendments***	**Description of Incentives**
MK-3102-011	0512	Taiwan	Hung, Yi-Jen<br>Tri-Service General Hospital<br>Division of Endocrinology and Metabolism<br>No.325, Sec.2, Chenggong Rd<br>Neihu District, Taipei City 114, (R.O.C)<br>Taiwan	He, Chih-Tsueng<br>Hsieh, Chang-Hsun<br>Lee, Chien-Hsing	3	Institutional Review Board<br>Room 5113, 5F, Medical building<br>No.325, Sec.2, Chenggong Rd<br>Neihu District, Taipei City 114, (R.O.C)<br>Taiwan	Yu, Mu-Hsien	Prot. 00 – 02-May-2013<br>Prot. Am. 02 – 02-Oct-2013<br>Prot. Am. 03 – 09-May-2014	None
MK-3102-011	0515	Taiwan	Lee, Ting-I<br>Taipei Medical University-Wan<br>Clinical Research Center, 14th Floor, Medical Building,<br>111, Sec. 3, Hsing-Ling Rd<br>Taipei 116,<br>Taiwan	Lee, Ting-Wei	0	TMU-Joint Institutional Review Board<br>250, Wu Hsing Street<br>Taipei 11031<br>Taiwan	Hsueh, Jui-Yuan	Prot. 00 – 14-May-2013<br>Prot. Am. 02 – 03-Sep-2013<br>Prot. Am. 03 – 02-Jul-2014	None
MK-3102-011	0516	Taiwan	Huang , Chen-Ling<br>Taipei Medical University Hospital<br>Clinical Research Center, 5th Floor, 1st Medical Building,<br>252, Wu Hsing Street<br>Taipei 11031<br>Taiwan	Hsu, Chung-huei<br>Lin, Chih-Hung<br>Weng, Shuen-Fu	1	TMU-Joint Institutional Review Board<br>250, Wu Hsing Street<br>Taipei 11031<br>Taiwan	Hsueh, Jui-Yuan	Prot. 00 – 14-May-2013<br>Prot. Am. 02 – 03-Sep-2013<br>Prot. Am. 03 – 02-Jul-2014	None
MK-3102-011	0518	Taiwan	Shin, Shyi-Jang<br>Next to Renal Function Room<br>5th Floor, No.100<br>Tzyou 1st Road<br>Kaohsiung 80756<br>Taiwan	Lin, Kun-Der<br>Li, Yu-Li	3	Kaohsiung Medical University Chung-Ho Memorial Hospital,<br>Institutional Review Board<br>No. 100, Tzyou 1st Road<br>Kaohsiung 807<br>Taiwan	Yen, Hsueh-Wei	Prot. 00 – 26-Sep-2013<br>Prot. Am. 02 – 10-Jan-2014<br>Prot. Am. 03 – 12-Sep-2014	None

Supplementary Table S5. IRB approval number and dates Study #24

Trial Number	Site Number	Country	Primary Investigator<br>Site Address	Sub-Investigators	Number of Subjects Enrolled	Name and Address of IEC	Chairperson (if known)	Date of IEC Approvals of Protocol(s) & Amendments<br>(Non-US, Non-IND Trial Sites)	ICF Incentives<br>(Non-US, Non-IND Trial Sites)
MK-3102-024	0001	United States	Lubin, Barry<br>National Clinical Research- Norfolk, Inc<br>885 Kempsville Road, Suite #221<br>Norfolk, VA 23502<br>United States	Beyer, Gail<br>Goldman, Charles	4	Schulman Associates IRB<br>4445 Lake Forest Drive, Suite 300<br>Cincinnati, OH 45242<br>United States	Nelson, Sharon	Prot. 00 - 05 Dec 2012<br>Prot. Am. 01 - 16 Feb 2013<br>Prot. Am. 04 - 28 Jun 2013<br>Prot. Am. 05 - 26 Mar 2014	Not applicable
MK-3102-024	0002	United States	Molter, Darron<br>North Myrtle Beach Family Practice<br>86 Cedar Avenue<br>North Myrtle Beach, SC 29582<br>United States	Ramsbottom, John	1	Schulman Associates IRB<br>4445 Lake Forest Drive, Suite 300<br>Cincinnati, OH 45242<br>United States	Nelson, Sharon	Prot. 00 - 05 Dec 2012<br>Prot. Am. 01 - 16 Feb 2013<br>Prot. Am. 04 - 28 Jun 2013<br>Prot. Am. 05 - 26 Mar 2014	Not applicable
MK-3102-024	0003	United States	Mosca, Robert /Ricci, Donato<br>Elite Research Network<br>3635 S. Clyde Morris Blvd. Suite 800<br>Port Orange, FL 32129<br>United States	Agnone, Louis<br>Moulis, Harry<br>Pasricha, Sunil<br>Patel, Ketul<br>Stella, Gregory	2	Schulman Associates IRB<br>4445 Lake Forest Drive, Suite 300<br>Cincinnati, OH 45242<br>United States	Nelson, Sharon	Prot. 00 - 05 Dec 2012<br>Prot. Am. 01 - 16 Feb 2013<br>Prot. Am. 04 - 28 Jun 2013<br>Prot. Am. 05 - 26 Mar 2014	Not applicable
MK-3102-024	0004	United States	Septimus, Joshua<br>Associates in Medicine, PA<br>4543 Post Oak Place Suite 105<br>Houston, TX 77027<br>United States	Jackson, Richard A.<br>Lisse, Scott<br>Niefield, Stewart<br>Posey, John A<br>Rubin, Shari<br>Sheena, Ronnie	3	Schulman Associates IRB<br>4445 Lake Forest Drive, Suite 300<br>Cincinnati, OH 45242<br>United States	Nelson, Sharon	Prot. 00 - 05 Dec 2012<br>Prot. Am. 01 - 16 Feb 2013<br>Prot. Am. 04 - 28 Jun 2013<br>Prot. Am. 05 - 26 Mar 2014	Not applicable
MK-3102-024	0005	United States	Trippett, J Mark<br>Sugar Lakes Family Practice<br>1327 Lake Pointe Parkway Suite 500<br>Sugar Land, TX 77478<br>United States	None	11	Schulman Associates IRB<br>4445 Lake Forest Drive, Suite 300<br>Cincinnati, OH 45242<br>United States	Nelson, Sharon	Prot. 00 - 05 Dec 2012<br>Prot. Am. 01 - 16 Feb 2013<br>Prot. Am. 04 - 28 Jun 2013<br>Prot. Am. 05 - 26 Mar 2014	Not applicable
MK-3102-024	0006	United States	Larsen, David<br>Wasatch Clinical Research<br>4001 South 700 East Suite 105<br>Salt Lake City, UT 84107<br>United States	Daynes, Randall<br>Fuller, Gene<br>Rasmussen, Brian<br>Young, Timothy	18	Schulman Associates IRB<br>4445 Lake Forest Drive, Suite 300<br>Cincinnati, OH 45242<br>United States	Nelson, Sharon	Prot. 00 - 05 Dec 2012<br>Prot. Am. 01 - 16 Feb 2013<br>Prot. Am. 04 - 28 Jun 2013<br>Prot. Am. 05 - 26 Mar 2014	Not applicable
MK-3102-024	0007	United States	Lewin, Andrew/Frias, Juan<br>National Research Institute<br>2010 Wilshire Blvd Suite 302<br>Los Angeles, CA 90057<br>United States	Hsia, Stanley HW<br>Kelly, Sharon<br>Nadkarni, Salil<br>Sandoval, Rose	30	Schulman Associates IRB<br>4445 Lake Forest Drive, Suite 300<br>Cincinnati, OH 45242<br>United States	Nelson, Sharon	Prot. 00 - 05 Dec 2012<br>Prot. Am. 01 - 16 Feb 2013<br>Prot. Am. 04 - 28 Jun 2013<br>Prot. Am. 05 - 26 Mar 2014	Not applicable
MK-3102-024	0008	United States	Hill, Gordon<br>Oakwell Clinical Research, LLC<br>3338 Oakwell Ct Ste 107 &110<br>San Antonio, TX 78218-3087<br>United States	Alprin, Clifford<br>Beck, Joshua<br>Sandercock, David<br>Walthall, Walter	13	Schulman Associates IRB<br>4445 Lake Forest Drive, Suite 300<br>Cincinnati, OH 45242<br>United States	Nelson, Sharon	Prot. 00 - 05 Dec 2012<br>Prot. Am. 01 - 16 Feb 2013<br>Prot. Am. 04 - 28 Jun 2013<br>Prot. Am. 05 - 26 Mar 2014	Not applicable
MK-3102-024	0009	United States	Resnick, Harvey<br>R/D Clinical Research, Inc.<br>461 This Way<br>Lake Jackson, TX 77566<br>United States	Feaver, Brian<br>Fuchs, Mary<br>Gambrel, Michele	0	Schulman Associates IRB<br>4445 Lake Forest Drive, Suite 300<br>Cincinnati, OH 45242<br>United States	Nelson, Sharon	Prot. 00 - 05 Dec 2012<br>Prot. Am. 01 - 16 Feb 2013<br>Prot. Am. 04 - 28 Jun 2013<br>Prot. Am. 05 - 26 Mar 2014	Not applicable
MK-3102-024	0010	United States	Canadas, Rafael<br>Galenos Research<br>12200 Park Central Drive Suite 200<br>Dallas, TX 75251<br>United States	Day, Gustavo H.	8	Schulman Associates IRB<br>4445 Lake Forest Drive, Suite 300<br>Cincinnati, OH 45242<br>United States	Nelson, Sharon	Prot. 00 - 05 Dec 2012<br>Prot. Am. 01 - 16 Feb 2013<br>Prot. Am. 04 - 28 Jun 2013<br>Prot. Am. 05 - 26 Mar 2014	Not applicable
MK-3102-024	0011	United States	Rovner, Sergio<br>10525 Vista del Sol Dr. Suite 210<br>El Paso, TX 79925<br>United States	Stewart-Ray, Verlaine	6	Schulman Associates IRB<br>4445 Lake Forest Drive, Suite 300<br>Cincinnati, OH 45242<br>United States	Nelson, Sharon	Prot. 00 - 05 Dec 2012<br>Prot. Am. 01 - 16 Feb 2013<br>Prot. Am. 04 - 28 Jun 2013<br>Prot. Am. 05 - 26 Mar 2014	Not applicable
MK-3102-024	0012	United States	Daniel, Jerome<br>Clinical Research Partners, LLC<br>1467 Johnston Willis Drive<br>Richmond, VA 23235<br>United States	James, Joseph	0	Schulman Associates IRB<br>4445 Lake Forest Drive, Suite 300<br>Cincinnati, OH 45242<br>United States	Nelson, Sharon	Prot. 00 - 05 Dec 2012<br>Prot. Am. 01 - 16 Feb 2013<br>Prot. Am. 04 - 28 Jun 2013<br>Prot. Am. 05 - 26 Mar 2014	Not applicable
MK-3102-024	0013	United States	Gabra, Nashwa<br>Burke Internal Medicine & Research<br>9243 Old Keen Mill Rd<br>Burke, VA 22015<br>United States	Andrawis, Nabil	7	Schulman Associates IRB<br>4445 Lake Forest Drive, Suite 300<br>Cincinnati, OH 45242<br>United States	Nelson, Sharon	Prot. 00 - 05 Dec 2012<br>Prot. Am. 01 - 16 Feb 2013<br>Prot. Am. 04 - 28 Jun 2013<br>Prot. Am. 05 - 26 Mar 2014	Not applicable
MK-3102-024	0014	United States	Mayfield, Ronald<br>Mountain View Clinical Research<br>405 Memorial Drive Extension<br>Greer, SC 29651-1817<br>United States	Ballard, Thomas<br>Byars, William<br>Durham, Nancy<br>Wood, Ruth	12	Schulman Associates IRB<br>4445 Lake Forest Drive, Suite 300<br>Cincinnati, OH 45242<br>United States	Nelson, Sharon	Prot. 00 - 05 Dec 2012<br>Prot. Am. 01 - 16 Feb 2013<br>Prot. Am. 04 - 28 Jun 2013<br>Prot. Am. 05 - 26 Mar 2014	Not applicable
MK-3102-024	0015	United States	Selam, Jean-Louis<br>University Clinical Investigators, Inc. dba Diabetes Research<br>2492 Walnut Ave Ste 130<br>Tustin, CA 92780-6953<br>United States	Van, Joanna	12	Schulman Associates IRB<br>4445 Lake Forest Drive, Suite 300<br>Cincinnati, OH 45242<br>United States	Nelson, Sharon	Prot. 00 - 05 Dec 2012<br>Prot. Am. 01 - 16 Feb 2013<br>Prot. Am. 04 - 28 Jun 2013<br>Prot. Am. 05 - 26 Mar 2014	Not applicable
MK-3102-024	0016	United States	Pimentel, Severino<br>West Memorial Family Practice<br>21304 Provincial Blvd<br>Katy, TX 77450<br>United States	Mould, Erin	1	Schulman Associates IRB<br>4445 Lake Forest Drive, Suite 300<br>Cincinnati, OH 45242<br>United States	Nelson, Sharon	Prot. 00 - 05 Dec 2012<br>Prot. Am. 01 - 16 Feb 2013<br>Prot. Am. 04 - 28 Jun 2013<br>Prot. Am. 05 - 26 Mar 2014	Not applicable
MK-3102-024	0017	United States	Blair-Britt, Loray/Luna, Blanca<br>Century Clinical Research, Inc<br>1410 LPGA Blvd Suite 132<br>Daytona Beach, FL 32117<br>United States	Gaskin, Tina	4	Schulman Associates IRB<br>4445 Lake Forest Drive, Suite 300<br>Cincinnati, OH 45242<br>United States	Nelson, Sharon	Prot. 00 - 05 Dec 2012<br>Prot. Am. 01 - 16 Feb 2013<br>Prot. Am. 04 - 28 Jun 2013<br>Prot. Am. 05 - 26 Mar 2014	Not applicable

Trial Number	Site Number	Country	Primary Investigator<br>Site Address	Sub-Investigators	Number of Subjects Enrolled	Name and Address of IEC	Chairperson (if known)	Date of IEC Approvals of Protocol(s) & Amendments (Non-US, Non-IND Trial Sites)	ICF Incentives (Non-US, Non-IND Trial Sites)
MK-3102-024	0019	United States	Miller, Sam<br>SAM Clinical Research Center<br>7711 Louis Pasteur Drive, Suite 300<br>San Antonio, TX 78229<br>United States	Carry, Rodney	9	Schulman Associates IRB<br>4445 Lake Forest Drive, Suite 300<br>Cincinnati, OH 45242<br>United States	Nelson, Sharon	Prot. 00 - 05 Dec 2012<br>Prot. Am. 01 - 16 Feb 2013<br>Prot. Am. 04 - 28 Jun 2013<br>Prot. Am. 05 - 26 Mar 2014	Not applicable
MK-3102-024	0020	United States	Zaky, Joseph<br>St. Mark Clinical Trials, LLC<br>7648 Seville Ave<br>Huntington Park, CA 90255<br>United States	Mattar, Peter<br>Zaky, Amal	8	Schulman Associates IRB<br>4445 Lake Forest Drive, Suite 300<br>Cincinnati, OH 45242<br>United States	Nelson, Sharon	Prot. 00 - 05 Dec 2012<br>Prot. Am. 01 - 16 Feb 2013<br>Prot. Am. 04 - 28 Jun 2013<br>Prot. Am. 05 - 26 Mar 2014	Not applicable
MK-3102-024	0201	Bulgaria	Nikitov, Zahari<br>MHAT Prof. Dr. Stoqn Kirkovich<br>11, Armeiska Str.<br>Stara Zagora 6000<br>Bulgaria	Nikolaeva, Antoaneta	4	Ethics Committee for Multicenter Trial<br>Sveta Nedelya' sqr., No: 5<br>Sofia, 1000<br>Bulgaria	Stoikov, Anastas	Prot. 00 - 03 Jun 2013<br>Prot. Am. 04 - 15 Jul 2013<br>Prot. Am. 05 - 02 Apr 2014	None
MK-3102-024	0202	Bulgaria	Yakov, Andrian<br>Mhat Gulyantsi<br>34, Vasil Levski Str.<br>Gulyantsi 5960<br>Bulgaria	Marinova, Temenuga	0	Ethics Committee for Multicenter Trial<br>Sveta Nedelya' sqr., No: 5<br>Sofia, 1000<br>Bulgaria	Stoikov, Anastas	Prot. 00 - 03 Jun 2013<br>Prot. Am. 04 - 15 Jul 2013<br>Prot. Am. 05 - 02 Apr 2014	None
MK-3102-024	0203	Bulgaria	Mitkov, Mitko<br>UMHAT "Sv.Georgi"<br>15A, Vassil Aprilov Blvd<br>Plovdiv 4002<br>Bulgaria	Nonchev, Boyan	8	Ethics Committee for Multicenter Trial<br>Sveta Nedelya' sqr., No: 5<br>Sofia, 1000<br>Bulgaria	Stoikov, Anastas	Prot. 00 - 03 Jun 2013<br>Prot. Am. 04 - 15 Jul 2013<br>Prot. Am. 05 - 02 Apr 2014	None
MK-3102-024	0204	Bulgaria	Levterov, Georgi<br>Multiprofile Hospital for Active Treatment (MHAT) – Kaspela<br>64, Sofia Str.<br>Plovdiv 4000<br>Bulgaria	Bojukliev, Atanas<br>Georgieva, Petya<br>Videnova, Elena<br>Yosifov, Milen<br>Zlatareva, Velichka	10	Ethics Committee for Multicenter Trial<br>Sveta Nedelya' sqr., No: 5<br>Sofia, 1000<br>Bulgaria	Stoikov, Anastas	Prot. 00 - 03 Jun 2013<br>Prot. Am. 04 - 15 Jul 2013<br>Prot. Am. 05 - 02 Apr 2014	None
MK-3102-024	0205	Bulgaria	Slavianov, Volen<br>Medical Center Teodora<br>101, Mutkurova Str.<br>Ruse 7000<br>Bulgaria	Bosilkov, Aleksander<br>Ivanov, Denislav<br>Petkov, Plamen<br>Stoyanov, Boris<br>Toncheva, Liliana	7	Ethics Committee for Multicenter Trial<br>Sveta Nedelya' sqr., No: 5<br>Sofia, 1000<br>Bulgaria	Stoikov, Anastas	Prot. 00 - 03 Jun 2013<br>Prot. Am. 04 - 15 Jul 2013<br>Prot. Am. 05 - 02 Apr 2014	None
MK-3102-024	0401	Hungary	Zilahi, Zsolt<br>Medifarma-98 Kft<br>Prága u. 9<br>Nyíregyháza 4400<br>Hungary	Nagy, Andrea	10	Egészségügyi Tudományos Tanács<br>Klinikai Farmakológiai Etikai Bizottsága<br>Arany János U. 6-9<br>Budapest H-1051<br>Hungary	Fürst, Zsuzsanna	Prot. 00 - 21 Mar 2013<br>Prot. Am. 04 - 27 Aug 2013<br>Prot Am. 05 - 30 Apr 2014	None
MK-3102-024	0402	Hungary	Wittmann, István<br>Pécsi Tudományegyetem<br>Pacsirta u 1.<br>Pécs 7624<br>Hungary	Kovács, Tibor	0	Egészségügyi Tudományos Tanács<br>Klinikai Farmakológiai Etikai Bizottsága<br>Arany János U. 6-9<br>Budapest H-1051<br>Hungary	Fürst, Zsuzsanna	Prot. 00 - 21 Mar 2013<br>Prot. Am. 04 - 27 Aug 2013<br>Prot Am. 05 - 30 Apr 2014	None
MK-3102-024	0404	Hungary	Vasas, Szilard<br>Borbánya Praxis Egeszségügyii Kft.<br>Bazsalikom str 1/1<br>Nyiregyhaza 4405<br>Hungary	Gégény, Enikő	14	Egészségügyi Tudományos Tanács<br>Klinikai Farmakológiai Etikai Bizottsága<br>Arany János U. 6-9<br>Budapest H-1051<br>Hungary	Fürst, Zsuzsanna	Prot. 00 - 21 Mar 2013<br>Prot. Am. 04 - 27 Aug 2013<br>Prot Am. 05 - 30 Apr 2014	None
MK-3102-024	0602	Mexico	Alpizar Salazar, Melchor<br>Centro Especializado en Diabetes, Obesidad y Prevencion de E<br>Calle 3 Col. Reforma Social<br>Mexico 11650<br>Mexico	Crespo Hernandez, Mariana	8	Comite de Etica en Investigacion del centro especializado en Diabetes y Obesidd y prevención de enfermedades cardiovasculares SC<br>Calle 3 No. 7 col. Reforma Social<br>Mexico 11650<br>Mexico	Ibañez, Armando Davalos	Prot Am. 04 - 21 Aug 2013<br>Prot Am. 05 - 25 Jun 2014	None
MK-3102-024	0801	Poland	Biniszkiewicz, Tomasz<br>Szpital Specjalistyczny nr 2 w Bytomiu<br>Ul. Stefana Batorego 15<br>Bytom 41-902<br>Poland	Bilska, Aleksandra<br>Mostowy, Aleksandra<br>Romanczyk, Adam	5	Komisja Bioetyczna przy Okregowej Izbie Lekarskiej<br>ul. Sniadeckich 33<br>Gdansk, 80-204<br>Poland	Umiastowski, Jerzy	Prot. 00 - 14 May 2013<br>Prot. Am. 04 - 17 Sep 2013<br>Prot. Am. 05 - 01 Apr 2014	None
MK-3102-024	0802	Poland	Majcher - Witczak, Grazyna<br>NZOZ Witamed<br>ul. Legionów 3/6<br>Kielce 25-035<br>Poland	Majsterkiewicz, Malgorzata<br>Matysiak, Elzbieta	2	Komisja Bioetyczna przy Okregowej Izbie Lekarskiej<br>ul. Sniadeckich 33<br>Gdansk, 80-204<br>Poland	Umiastowski, Jerzy	Prot. 00 - 14 May 2013<br>Prot. Am. 04 - 17 Sep 2013<br>Prot. Am. 05 - 01 Apr 2014	None
MK-3102-024	0803	Poland	Sroka, Michal<br>Wojewodzki Szpital Specjalist. we Wroclawiu<br>ul. H. Kamienskiego 73ª<br>Wroclaw 51-124<br>Poland	Guziewicz, Malgorzata	1	Komisja Bioetyczna przy Okregowej Izbie Lekarskiej<br>ul. Sniadeckich 33<br>Gdansk, 80-204<br>Poland	Umiastowski, Jerzy	Prot. 00 - 14 May 2013<br>Prot. Am. 04 - 17 Sep 2013<br>Prot. Am. 05 - 01 Apr 2014	None
MK-3102-024	0804	Poland	Franek, Edward<br>Centralny Szpital Kliniczny MSW w Warszawie<br>Ul. Woloska 137<br>Warszawa 02-507<br>Poland	Jedynasty, Krystyna<br>Lesniewski, Robert	11	Komisja Bioetyczna przy Okregowej Izbie Lekarskiej<br>ul. Sniadeckich 33<br>Gdansk, 80-204<br>Poland	Umiastowski, Jerzy	Prot. 00 - 14 May 2013<br>Prot. Am. 04 - 17 Sep 2013<br>Prot. Am. 05 - 01 Apr 2014	None
MK-3102-024	0805	Poland	Krzyzagorska, Ewa<br>Praktyka Lekarska Ewa Krzyzagorska<br>ul. Murawa 37ª<br>Poznan 61-655<br>Poland	Sekulska, Marzenna	4	Komisja Bioetyczna przy Okregowej Izbie Lekarskiej<br>ul. Sniadeckich 33<br>Gdansk, 80-204<br>Poland	Umiastowski, Jerzy	Prot. 00 - 14 May 2013<br>Prot. Am. 04 - 17 Sep 2013<br>Prot. Am. 05 - 01 Apr 2014	None
MK-3102-024	0806	Poland	Semetkowska - Jurkiewicz, Ewa<br>ISPL Centrum Medyczne<br>ul.Rajska 10 lok.6<br>Gdańsk 80-850<br>Poland	Jaromczyk - Slisz, Janina	3	Komisja Bioetyczna przy Okregowej Izbie Lekarskiej<br>ul. Sniadeckich 33<br>Gdansk, 80-204<br>Poland	Umiastowski, Jerzy	Prot. 00 - 14 May 2013<br>Prot. Am. 04 - 17 Sep 2013<br>Prot. Am. 05 - 01 Apr 2014	travel reimbursement

Trial Number	Site Number	Country	Primary Investigator<br>Site Address	Sub-Investigators	Number of Subjects Enrolled	Name and Address of IEC	Chairperson (if known)	Date of IEC Approvals of Protocol(s) & Amendments<br>(Non-US, Non-IND Trial Sites)	ICF Incentives<br>(Non-US, Non-IND Trial Sites)
MK-3102-024	0901	Romania	Bala, Cornelia Gabriela<br>Spitalul Clinic Judetean de Urgenta Cluj<br>Str. Clinicilor, nr. 2-4<br>Cluj-Napoca 400006<br>Romania	Cerghizan, Anca<br>Duma, Livia Terezia<br>Roman, Gabriela<br>Veresiu, Ioan Andrei	0	Comisia de Bioetica<br>Sos. Stefan cel Mare nr.19-21<br>Bucuresti 020125<br>Romania	Dumitrescu, Sava	Prot. 00 - 09 Jul 2013<br>Prot. Am. 04 - 15 Jan 2014<br>Prot Am. 05 - 03 Apr 2014	None
MK-3102-024	0903	Romania	Cif, Adriana<br>Mediab<br>Str. Gheorghe Marinescu nr. 8A, ap II<br>Targu-Mures 540142<br>Romania	Suciu, Elena Mirela	9	Comisia de Bioetica<br>Sos. Stefan cel Mare nr.19-21<br>Bucuresti 020125<br>Romania	Dumitrescu, Sava	Prot. 00 - 09 Jul 2013<br>Prot. Am. 04 - 15 Jan 2014<br>Prot Am. 05 - 03 Apr 2014	None
MK-3102-024	0904	Romania	Gagiu, Remus<br>Gagiu D. Remus - Cabinet Medical Individual<br>Str. Tudor Vladimirescu nr. 39, Bloc 2,<br>Targoviste 130083<br>Romania	Gagiu, Andreea	12	Comisia de Bioetica<br>Sos. Stefan cel Mare nr.19-21<br>Bucuresti 020125<br>Romania	Dumitrescu, Sava	Prot. 00 - 09 Jul 2013<br>Prot. Am. 04 - 15 Jan 2014<br>Prot Am. 05 - 03 Apr 2014	None
MK-3102-024	0905	Romania	Pintilei, Ella<br>Consultmed<br>Str. Pacurari nr. 70, Bloc 550<br>Iasi 700514<br>Romania	Busuioc-Witowschi, Dan<br>Ganga, Mihai	2	Comisia de Bioetica<br>Sos. Stefan cel Mare nr.19-21<br>Bucuresti 020125<br>Romania	Dumitrescu, Sava	Prot. 00 - 09 Jul 2013<br>Prot. Am. 04 - 15 Jan 2014<br>Prot Am. 05 - 03 Apr 2014	None
MK-3102-024	0906	Romania	Popa, Bogdan<br>Spitalul Judetean de Urgente Ploiesti<br>Strada Gageni 100<br>Ploiesti 100097<br>Romania	Porojnicu, Catalina	5	Comisia de Bioetica<br>Sos. Stefan cel Mare nr.19-21<br>Bucuresti 020125<br>Romania	Dumitrescu, Sava	Prot. 00 - 09 Jul 2013<br>Prot. Am. 04 - 15 Jan 2014<br>Prot Am. 05 - 03 Apr 2014	None
MK-3102-024	0907	Romania	Barbonta, Diana<br>Str. Closca, no.6 bl. 4, Sc. F, Ap. 70<br>Alba Iulia 510053<br>Romania	Barbonta, Cristian Gelu<br>Suleac, Oana	8	Comisia de Bioetica<br>Sos. Stefan cel Mare nr.19-21<br>Bucuresti 020125<br>Romania	Dumitrescu, Sava	Prot. 00 - 09 Jul 2013<br>Prot. Am. 04 - 15 Jan 2014<br>Prot Am. 05 - 03 Apr 2014	None
MK-3102-024	0908	Romania	Creteanu, Gabriela Ileana<br>Spitalul Judetean de Urgenta "Sf. Ioan cel Nou"<br>Bd. 1 Decembrie 1918 nr. 21<br>Suceava 720237<br>Romania	Caziuc, Ramona Camelia<br>Clus, Simona	2	Comisia de Bioetica<br>Sos. Stefan cel Mare nr.19-21<br>Bucuresti 020125<br>Romania	Dumitrescu, Sava	Prot. 00 - 09 Jul 2013<br>Prot. Am. 04 - 15 Jan 2014<br>Prot Am. 05 - 03 Apr 2014	None
MK-3102-024	0909	Romania	Zetu, Cornelia<br>Inst Nat de Diab, Nutritie si Boli Metabolice NC Paulescu<br>Str.Ion Movila nr.5-7<br>Bucuresti 020475<br>Romania	Dondoi, Carmen<br>Tapurica, Minodora	10	Comisia de Bioetica<br>Sos. Stefan cel Mare nr.19-21<br>Bucuresti 020125<br>Romania	Dumitrescu, Sava	Prot. 00 - 09 Jul 2013<br>Prot. Am. 04 - 15 Jan 2014<br>Prot Am. 05 - 03 Apr 2014	None
MK-3102-024	1001	Russian Federation	Abrosimov, Vladimir<br>Ryazan Regional Clinical Hospital for war's veterans<br>Str. Semashko, 3<br>Ryazan 390005<br>Russian Federation	Glotov, Sergei<br>Ponomareva, Irina<br>Zhuravleva, Natalya	2	The Ethics Council under Ministry of Health of Russian Federation<br>3. Rakhmanovsky pereulok<br>Moscow<br>Russian Federation	Chuchalin, Alexandr	Prot. 00 - 24 Jun 2013<br>Prot. Am. 04 - 13 Aug 2013<br>Prot. Am. 05 - 30 Apr 2013	None
MK-3102-024	1002	Russian Federation	Yakusevich, Vladimir<br>State Clinical Hospital for Emergency Medical Care n.a.N.V.<br>11, Zagorodny sad<br>Yaroslavl 150003<br>Russian Federation	Kabanov, Andrey<br>Konoplina, Olga<br>Orekhova, Irina<br>Stroganova, Elena<br>Svetalkina, Ekaterina	3	The Ethics Council under Ministry of Health of Russian Federation<br>3. Rakhmanovsky pereulok<br>Moscow<br>Russian Federation	Chuchalin, Alexandr	Prot. 00 - 24 Jun 2013<br>Prot. Am. 04 - 13 Aug 2013<br>Prot. Am. 05 - 30 Apr 2013	None
MK-3102-024	1003	Russian Federation	Mustafina, Svetlana<br>LLC Reafan<br>175/1, Borisa Bogatkova ul.<br>Novosibirsk 630089<br>Russian Federation	Karnaukhov, Vitaly<br>Kondratova, Maria<br>Peshkov, Eugenij<br>Rybina, Olga<br>Tuguleva, Tatiana<br>Zhukova, Julia	7	The Ethics Council under Ministry of Health of Russian Federation<br>3. Rakhmanovsky pereulok<br>Moscow<br>Russian Federation	Chuchalin, Alexandr	Prot. 00 - 24 Jun 2013<br>Prot. Am. 04 - 13 Aug 2013<br>Prot. Am. 05 - 30 Apr 2013	None
MK-3102-024	1004	Russian Federation	Obrezan, Andrey<br>LLC"International medical center "SOGAZ"<br>8A, Malaya Konushennaya<br>Saint-Petersburg 191168<br>Russian Federation	Tcvetkova, Svetlana<br>Melnikova, Maria	0	The Ethics Council under Ministry of Health of Russian Federation<br>3. Rakhmanovsky pereulok<br>Moscow<br>Russian Federation	Chuchalin, Alexandr	Prot. 00 - 24 Jun 2013<br>Prot. Am. 04 - 13 Aug 2013<br>Prot. Am. 05 - 30 Apr 2013	None
MK-3102-024	1005	Russian Federation	Verbovaya, Nelly<br>LLC "Center Diabetes"<br>Sovetskoy Armii 56<br>Samara 443067<br>Russian Federation	Verbovaya, Maria<br>Barabanova, Natalia<br>Pashentseva, Anna<br>Scudaev, Sergey<br>Suldina, Tatiana	9	The Ethics Council under Ministry of Health of Russian Federation<br>3. Rakhmanovsky pereulok<br>Moscow<br>Russian Federation	Chuchalin, Alexandr	Prot. 00 - 24 Jun 2013<br>Prot. Am. 04 - 13 Aug 2013<br>Prot. Am. 05 - 30 Apr 2013	None
MK-3102-024	1006	Russian Federation	Fitilev, Sergey<br>GBUZ Municipal Clinic #2 of Healthcare Department of Moscow<br>12 Fruktovaya str.<br>Moscow 117556<br>Russian Federation	Vozzhaev, Aleksandr<br>Chernobaeva, Nelly<br>Karmanova, Svetlana<br>Shkrebniova, Irina<br>Yakushev, Vadim	1	The Ethics Council under Ministry of Health of Russian Federation<br>3. Rakhmanovsky pereulok<br>Moscow<br>Russian Federation	Chuchalin, Alexandr	Prot. 00 - 24 Jun 2013<br>Prot. Am. 04 - 13 Aug 2013<br>Prot. Am. 05 - 30 Apr 2013	None
MK-3102-024	1101	Slovakia	Schroner, Zbynek<br>SchronerMED, s.r.o.<br>Ceskoslovenskej Armady 35<br>Moldava nad Bodvou 045 25<br>Slovakia	Schronerova, Monika	7	Eticka komisia Kosickeho samospravneho kraja<br>Namestie Maratonu mieru 1<br>Kosice, 042 66<br>Slovakia (CEC)	Grullingova, Maria	Prot. 00 - 26 Feb 2013<br>Prot. Am. 04 - 16 Jul 2013<br>Prot. Am. 05 - 25 Mar 2014	None
MK-3102-024	1102	Slovakia	Merciakova, Martina<br>MEDI-DIA s.r.o<br>Namestie slobody 65<br>Sabinov 08301<br>Slovakia	Gasperikova, Gabriela	11	Eticka komisia Kosickeho samospravneho kraja<br>Namestie Maratonu mieru 1<br>Kosice, 042 66<br>Slovakia (CEC)	Grullingova, Maria	Prot. 00 - 26 Feb 2013<br>Prot. Am. 04 - 16 Jul 2013<br>Prot. Am. 05 - 25 Mar 2014	None
MK-3102-024	1103	Slovakia	Ilavska, Adriana<br>Medispektrum, s.r.o.<br>Gercenova 4/A<br>Bratislava 851 01<br>Slovakia	Kratky, Adrian	19	Eticka komisia Kosickeho samospravneho kraja<br>Namestie Maratonu mieru 1<br>Kosice, 042 66<br>Slovakia (CEC)	Grullingova, Maria	Prot. 00 - 26 Feb 2013<br>Prot. Am. 04 - 16 Jul 2013<br>Prot. Am. 05 - 25 Mar 2014	None

Trial Number	Site Number	Country	Primary Investigator<br>Site Address	Sub-Investigators	Number of Subjects Enrolled	Name and Address of IEC	Chairperson (if known)	Date of IEC Approvals of Protocol(s) & Amendments (Non-US, Non-IND Trial Sites)	ICF Incentives (Non-US, Non-IND Trial Sites)
MK-3102-024	1104	Slovakia	Buganova, Ingrid<br>MEDIVASA s.r.o.<br>Vojtecha Spanyola 8187<br>Zilina 010 01<br>Slovakia	Bugan, Viliam	5	Eticka komisia Kosickeho samospravneho kraja<br>Namestie Maratonu mieru 1<br>Kosice, 042 66<br>Slovakia (CEC)	Grullingova, Maria	Prot. 00 - 26 Feb 2013<br>Prot. Am. 04 - 16 Jul 2013<br>Prot. Am. 05 - 25 Mar 2014	None
MK-3102-024	1105	Slovakia	Ehsan, Noman<br>NOEMIS, s.r.o.<br>Dibrovova 26<br>Nove Mesto nad Vahom 915 01<br>Slovakia	Ehsanova, Monika	6	Eticka komisia Kosickeho samospravneho kraja<br>Namestie Maratonu mieru 1<br>Kosice, 042 66<br>Slovakia (CEC)	Grullingova, Maria	Prot. 00 - 26 Feb 2013<br>Prot. Am. 04 - 16 Jul 2013<br>Prot. Am. 05 - 25 Mar 2014	None
MK-3102-024	1106	Slovakia	Markova, Iveta<br>MUDr. Iveta Markova s.r.o.<br>Cajkovskeho 46<br>Nitra 949 11<br>Slovakia	Henzel, Zigmund	5	Eticka komisia Kosickeho samospravneho kraja<br>Namestie Maratonu mieru 1<br>Kosice, 042 66<br>Slovakia (CEC)	Grullingova, Maria	Prot. 00 - 26 Feb 2013<br>Prot. Am. 04 - 16 Jul 2013<br>Prot. Am. 05 - 25 Mar 2014	None
MK-3102-024	1107	Slovakia	Gabrisova, Alena<br>DIABETES CENTRUM, s.r.o.<br>K dolnej stanici 18<br>Trencin 911 01<br>Slovakia	Ondreickova, Zuzana	3	Eticka komisia Kosickeho samospravneho kraja<br>Namestie Maratonu mieru 1<br>Kosice, 042 66<br>Slovakia (CEC)	Grullingova, Maria	Prot. 00 - 26 Feb 2013<br>Prot. Am. 04 - 16 Jul 2013<br>Prot. Am. 05 - 25 Mar 2014	None
MK-3102-024	1201	South Africa	Abdullah, Ismail<br>Practice Abdullah<br>Gatesville Medical Centre Room 308, Third Floor<br>Cape Town 7764<br>South Africa	Bruning, Axel	0	Pharma-Ethics Independent Re<br>123 Amcor Road P.O. Box 786<br>Irene, 0062 Lyttelton Manor Gauteng 0157<br>South Africa	Duvenage. CSJ	Prot. 00 - 24 Jun 2013<br>Prot. Am. 04 - 17 Sep 2013<br>Prot. Am. 05 - 08 May 2014	None
MK-3102-024	1202	South Africa	Naidoo, Visvakuren<br>Practice Dr. V. Naidoo<br>8 Flamco Terrace Bayview<br>Chatsworth 4092<br>South Africa	Naidoo, Vievikka	1	Pharma-Ethics Independent Re<br>123 Amcor Road P.O. Box 786<br>Irene, 0062 Lyttelton Manor Gauteng 0157<br>South Africa	Duvenage. CSJ	Prot. 00 - 24 Jun 2013<br>Prot. Am. 04 - 17 Sep 2013<br>Prot. Am. 05 - 08 May 2014	None
MK-3102-024	1203	South Africa	Punt, Zelda<br>Phoenix Pharma (Pty) Ltd<br>2 Eastbourne Road Mount Croix<br>Port Elizabeth 6001<br>South Africa	Malan, Daniel	7	Pharma-Ethics Independent Re<br>123 Amcor Road P.O. Box 786<br>Irene, 0062 Lyttelton Manor Gauteng 0157<br>South Africa	Duvenage. CSJ	Prot. 00 - 24 Jun 2013<br>Prot. Am. 04 - 17 Sep 2013<br>Prot. Am. 05 - 08 May 2014	None
MK-3102-024	1204	South Africa	Sauermann, Lynette<br>Dr.'s Sauermann + Meyer<br>30b Jorissen Street<br>Polokwane 0699<br>South Africa	Meyer, Marti	0	Pharma-Ethics Independent Re<br>123 Amcor Road P.O. Box 786<br>Irene, 0062 Lyttelton Manor Gauteng 0157<br>South Africa	Duvenage. CSJ	Prot. 00 - 24 Jun 2013<br>Prot. Am. 04 - 17 Sep 2013<br>Prot. Am. 05 - 08 May 2014	None
MK-3102-024	1205	South Africa	Bhorat, As'ad<br>Soweto Clinical Trials Centre<br>1900 Sycamore Street Dlamini, Ext 2<br>Johannesburg 1818<br>South Africa	Bhorat, Qasim<br>Vorajee, Haroon	21	Pharma-Ethics Independent Re<br>123 Amcor Road P.O. Box 786<br>Irene, 0062 Lyttelton Manor Gauteng 0157<br>South Africa	Duvenage. CSJ	Prot. 00 - 24 Jun 2013<br>Prot. Am. 04 - 17 Sep 2013<br>Prot. Am. 05 - 08 May 2014	None
MK-3102-024	1207	South Africa	van der Walt, Eugene<br>Synexus SA - Roodeport Medicross<br>Roodepoort Medicross 54 Ontdekkers Road Roodepoort<br>Johannesburg 1724<br>South Africa	Smook, Anton<br>Steynberg, Willem	1	Pharma-Ethics Independent Re<br>123 Amcor Road P.O. Box 786<br>Irene, 0062 Lyttelton Manor Gauteng 0157<br>South Africa	Duvenage. CSJ	Prot. 00 - 24 Jun 2013<br>Prot. Am. 04 - 17 Sep 2013<br>Prot. Am. 05 - 08 May 2014	None
MK-3102-024	1208	South Africa	Naiker, Puvanesveri<br>Chelmsford Medical Centre 3<br>St. Augustine's Hospital Chelmsford Medical Centre 3<br>Durban 4001<br>South Africa	Nayiager, Savithree	4	Pharma-Ethics Independent Re<br>123 Amcor Road P.O. Box 786<br>Irene, 0062 Lyttelton Manor Gauteng 0157<br>South Africa	Duvenage. CSJ	Prot. 00 - 24 Jun 2013<br>Prot. Am. 04 - 17 Sep 2013<br>Prot. Am. 05 - 08 May 2014	None
MK-3102-024	1301	Spain	Jodar Gimeno, José Esteban<br>H.U. Quiron Madrid<br>Hospital Quirón Madrid. Endocrinologia y Nutrición. Diego de Velázquez, 1.<br>Planta -1<br>Pozuelo de Alarcón 28223<br>Spain	Pérez Arroyo, Maria Beatriz	1	H.U.P Hierro-Majadahonda<br>(Entrada por Laboratorios-Banco de Sangre) Planta 0 – al lado del pasillo de hospitalizaciones F C/ Joaquín Rodrigo, 2 Majadahonda Madrid 28222<br>Spain	Solá, Avendaño	Prot. 00 - 25 Mar 2013<br>Prot. Am. 04 - 24 Jun 2013<br>Prot. Am. 05 - 28 Apr 2014	None
MK-3102-024	1302	Spain	Martínez Navarro, Encarna<br>CAP Centelles - EAP Osona Sud-Alt Congost, S. L.<br>CAP Centelles - EAP Osona Sud-Alt Congost, S. L. Pla del Mestre, 7<br>Centelles 08540<br>Spain	Álvarez Sánchez, Maria Carmen<br>Narejos Perez, Silvia	0	H.U.P Hierro-Majadahonda<br>(Entrada por Laboratorios-Banco de Sangre) Planta 0 – al lado del pasillo de hospitalizaciones F C/ Joaquín Rodrigo, 2 Majadahonda Madrid 28222<br>Spain	Solá, Avendaño	Prot. 00 - 25 Mar 2013<br>Prot. Am. 04 - 24 Jun 2013<br>Prot. Am. 05 - 28 Apr 2014	None
MK-3102-024	1303	Spain	Garcia Ortiz, Luis<br>C.S. La Alamedilla<br>Centro de Salud La Alamedilla, Medicina General Avenida Comuneros 27-31<br>Salamanca 37003<br>Spain	Manuel Angel, Gómez Marcos,	2	H.U.P Hierro-Majadahonda<br>(Entrada por Laboratorios-Banco de Sangre) Planta 0 – al lado del pasillo de hospitalizaciones F C/ Joaquín Rodrigo, 2 Majadahonda Madrid 28222<br>Spain	Solá, Avendaño	Prot. 00 - 25 Mar 2013<br>Prot. Am. 04 - 24 Jun 2013<br>Prot. Am. 05 - 28 Apr 2014	None
MK-3102-024	1304	Spain	Ferrer Garcia, Juan Carlos<br>C.H.G.U. de Valencia<br>Consorcio Hospital General Universitario de Valencia. Servicio Endocrinología Avda. Tres Cruces, s/n<br>Valencia 46014<br>Spain	Sánchez Juan, Carlos	3	H.U.P Hierro-Majadahonda<br>(Entrada por Laboratorios-Banco de Sangre) Planta 0 – al lado del pasillo de hospitalizaciones F C/ Joaquín Rodrigo, 2 Majadahonda Madrid 28222<br>Spain	Solá, Avendaño	Prot. 00 - 25 Mar 2013<br>Prot. Am. 04 - 24 Jun 2013<br>Prot. Am. 05 - 28 Apr 2014	None
MK-3102-024	1305	Spain	Mezquita Raya, Pedro<br>Clínica San Pedro<br>Plaza San Pedro, 5-1 # 3<br>Almería 04001<br>Spain	None	0	H.U.P Hierro-Majadahonda<br>(Entrada por Laboratorios-Banco de Sangre) Planta 0 – al lado del pasillo de hospitalizaciones F C/ Joaquín Rodrigo, 2 Majadahonda Madrid 28222<br>Spain	Solá, Avendaño	Prot. 00 - 25 Mar 2013<br>Prot. Am. 04 - 24 Jun 2013<br>Prot. Am. 05 - 28 Apr 2014	None
MK-3102-024	1306	Spain	Pasquau Liaño, Francisco<br>H. Marina Baixa<br>Av. Jaume Botella Mayor, 7<br>Villajoyosa 03570<br>Spain	None	0	H.U.P Hierro-Majadahonda<br>(Entrada por Laboratorios-Banco de Sangre) Planta 0 – al lado del pasillo de hospitalizaciones F C/ Joaquín Rodrigo, 2 Majadahonda Madrid 28222<br>Spain	Solá, Avendaño	Prot. 00 - 25 Mar 2013<br>Prot. Am. 04 - 24 Jun 2013<br>Prot. Am. 05 - 28 Apr 2014	None
